# Preventing inappropriate signals pre- and post-ligand perception by a toggle switch mechanism of ERECTA

**DOI:** 10.1073/pnas.2420196122

**Published:** 2025-01-22

**Authors:** Liangliang Chen, Michal Maes, Alicia M. Cochran, Julian R. Avila, Paul Derbyshire, Jan Sklenar, Kelsey M. Haas, Judit Villén, Frank L.H. Menke, Keiko U. Torii

**Affiliations:** ^a^HHMI, The University of Texas at Austin, Austin, TX 78712; ^b^Department of Molecular Biosciences, The University of Texas at Austin, Austin, TX 78712; ^c^HHMI, University of Washington, Seattle, WA 98195; ^d^Department of Biology, University of Washington, Seattle, WA 98195; ^e^The Sainsbury Laboratory, University of East Anglia, Norwich Research Park, Norwich NR4 7UH, United Kingdom; ^f^Department of Genome Sciences, University of Washington, Seattle, WA 98195

**Keywords:** receptor kinase, phosphoswitch, peptide hormone, stomatal development, inflorescence growth

## Abstract

Dynamic control of signaling events requires swift regulation of receptors at an active state. By focusing on the Arabidopsis ERECTA (ER) receptor kinase, which perceives peptide ligands to control multiple developmental processes, we report a mechanism preventing inappropriate receptor activity. The ER C-terminal tail (ER_CT) functions as an autoinhibitory domain: Its removal confers higher kinase activity and hyperactivity during inflorescence and stomatal development. ER_CT is required for the binding of a receptor kinase inhibitor, BKI1, and two U-box E3 ligases, PUB30 and PUB31, that trigger activated ER to degradation through ubiquitination. We further identify ER_CT as a phosphodomain transphosphorylated by the coreceptor BAK1. The phosphorylation impacts the tail structure, likely releasing ER from autoinhibition. The phosphonull version enhances BKI1 association, whereas the phosphomimetic version promotes PUB30/31 association. Thus, ER_CT acts as an off–on–off toggle switch, facilitating the release of BKI1 inhibition, enabling signal activation, and swiftly turning over the receptors afterward. Our results elucidate a mechanism that fine-tunes receptor signaling via a phosphoswitch module, maintaining the receptor at a low basal state while ensuring robust yet transient activation upon ligand perception.

## Significance

Cells perceive and process external signals through their cell-surface receptors, whose activity must be tightly maintained to prevent the spread of misinformation. How do plant cells prevent inappropriate receptor activity? We identify a structural module within the C-terminal tail of the receptor-kinase ERECTA (ER_CT) that inhibits the receptor pre- and post-signal activation. The ER_CT comprises a linker and an α-Helix. Before activation, ER_CT is autoinhibitory and associates with an inhibitory protein. Ligand perception triggers the transphosphorylation of ER_CT by the coreceptor, which then recruits a degradation machinery to turn over the activated receptor swiftly. Thus, we reveal an off–on–off toggle switch mechanism that finely adjusts the activity of the plant receptor, enabling precise control over cell signaling.

Cell communication, both among individual cells and between cells and their environment, is fundamental to the survival and function of multicellular organisms, including higher plants. To manage this complex communication, plants have evolved an extensive superfamily of receptor-like kinases (RLKs), referred to hereafter as receptor kinases (RKs) for those with known functions. The RKs perceive and transmit external and endogenous signals, and their appropriate outputs modulate plant fitness as well as stress tolerance (1–5). A typical plant RLK contains an extracellular domain, a single transmembrane domain, and a cytoplasmic kinase domain. Based on various ectodomains, plant RLKs are classified into different subfamilies, among which those with extracellular leucine-rich repeat (LRR) domain, LRR-RLKs, comprise the largest subfamily (1, 6, 7). Primary LRR-RKs perceive signaling ligands, many of which are secreted peptides (8, 9). Well-studied examples of such LRR-RKs include the brassinosteroid (BR) receptor BRASSINOSTEROID INSENSITIVE1 (BRI1) and the pattern recognition receptor FLAGELLIN SENSING 2 (FLS2) (10, 11).

The early signaling events of receptor activation have been well-documented for BRI1 and FLS2 (12). In the absence of ligands, BRI1 is maintained in a basal state by its autoinhibitory C-terminal tail and BRI1 KINASE INHIBITOR 1 (BKI1), which associates with the kinase domain of BRI1 (13, 14). Specifically, the BRI1-interacting motif (BIM) within BKI1 serves as an interaction interface with the C-lobe of the BRI1 kinase domain (15). In addition, BKI1 plays a dual role in BR signaling by interacting with a subset of 14-3-3 proteins through the 14-3-3 binding motif (16, 17). Upon BR perception, BRI1 recruits its coreceptors, LRR-RKs from SOMATIC EMBRYOGENESIS RECEPTOR KINASES (SERKs) family (including BRI1-ASSOCIATED KINASE1 (BAK1)/SERK3, SERK1, and SERK4) via its extracellular LRR domain and disassociates with BKI1 to trigger the phosphorylation events within the BRI1-BAK1/SERK complex (18–20). In vitro and in vivo phosphorylation analyses have shown sequential auto- and transphosphorylation events between BRI1 and BAK1 (14, 21). Likewise, rapid heterodimerization and phosphorylation ensue between FLS2 and BAK1 upon perception of a flagellin peptide flg22 (22). Further phosphorylation analyses of BAK1/SERKs provided insight into specific phosphorylation signatures likely associated with developmental *vs.* immune signaling (23, 24). BAK1 and SERKs function as coreceptors for many other LRR-RKs and play crucial roles in plant growth and development, as well as plant immunity (20). Following the activation of receptor complexes, the signal will be attenuated through posttranslational modifications of the activated receptor kinases. The downregulation of receptor signaling is crucial to fine-tune signaling outputs. For example, two closely related plant U-box (PUB) E3 ubiquitin ligases, PUB12 and PUB13, engage in the ligand-induced ubiquitination and degradation for both BRI1 and FLS2 (25, 26). However, the specific domain within these LRR-RKs that recruits PUB12/13 remains unclear.

The three Arabidopsis ER-family LRR-RKs (ER, ER-LIKE1 (ERL1), and ERL2) regulate many developmental processes, including shoot meristem homeostasis, leaf serration, inflorescence elongation, flower and ovule development, vascular differentiation, and stomatal patterning (27–35). Particularly, loss-of-function mutations in *ER* result in excessive asymmetric cell divisions during stomatal development, as well as a compact inflorescence with short pedicels (33–35). The activity of ER-family receptors is regulated by cysteine-rich secreted peptides from the EPIDERMAL PATTERNING FACTOR (EPF)/EPF-LIKE (EPFL) family: EPF2 and EPF1 are primarily perceived by ER and ERL1, respectively, to inhibit stomatal development (36–39). In contrast, EPFL9 (also known as Stomagen) promotes stomatal development as an antagonist of EPF2 and EPF1 through competitively binding to ER and ERL1, respectively (40–42). Moreover, in developing stems, two other EPF/EPFL family peptides, EPFL4 and EPFL6, also activate ER to promote inflorescence elongation (43). It remains unknown how the ER kinase activity is regulated before the ligand perception.

Perception of EPF/EPFL peptides triggers the heterodimerization of ER with its coreceptor, BAK1/SERKs, which causes transphosphorylation between the ER and SERK family coreceptors (44). After signal activation, PUB30 and PUB31 are phosphorylated by BAK1 and subsequently ubiquitinate the ligand-activated ER, leading to its eventual degradation (45). Neither the exact sites of transphosphorylation of ER by BAK1 nor how such phosphorylation events trigger the recruitment of PUB30/31 are known. Site-directed mutagenesis studies of the ER kinase domain, including those putative phosphorylation sites within the activation loop, have been performed before (23, 28). Yet, these studies failed to reveal the actual phosphorylation events signifying the receptor activation and/or inactivation.

Here, we report that the ER C-terminal tail, ER_CT, functions as a phosphorylation-controlled three-way off–on–off toggle switch to ensure the proper window of ER signal activation. The ER_CT possesses a characteristic α-Helix and is deeply conserved through the ER orthologs and paralogs. Its removal confers receptor hyperactivity, triggers excessive inflorescence elongation, and overinhibition of the stomatal cell lineages. The ER_CT is essential for the binding of the negative regulators, BKI1 and PUB30/31. Using mass spectrometry, we demonstrate that BAK1 transphosphorylates ER and that the phosphonull version of ER_CT (inactive state) associates with BKI1 whereas the phosphomimetic version (activated state) associates with PUB30/31. Our study elucidates the role of the ER_CT phosphomodule to limit the window of receptor activation for properly fine-tuning the signal outcome and provide further insight into the modes of action of plant receptor kinases.

## Results

### ER-Family Receptor Kinases Possess a Long Conserved C-Terminal Tail.

**Fig. 1.**
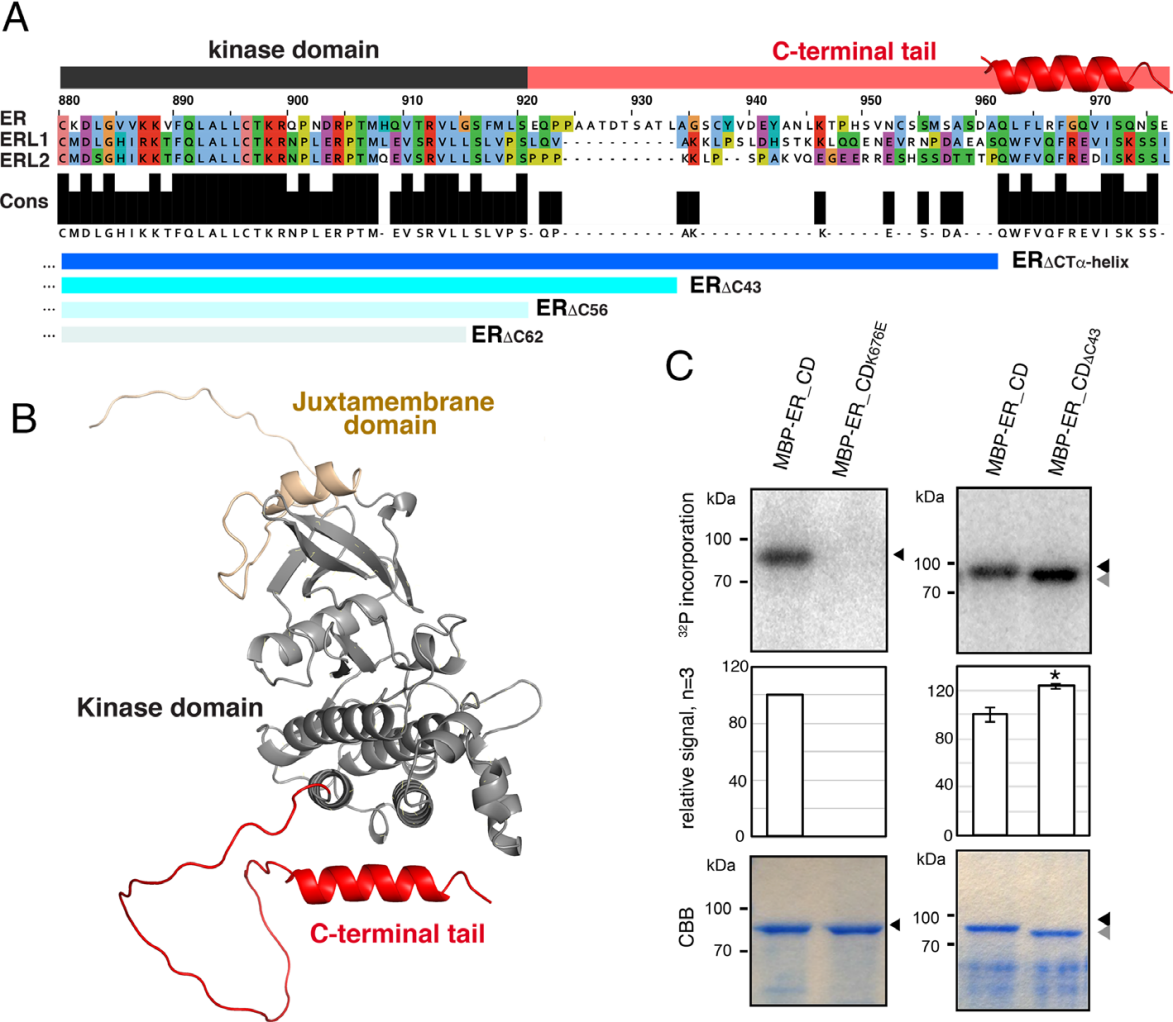
The conserved C-terminal tail in ER-family LRR-RKs inhibits the kinase activity. (*A*) Alignment of the C-terminal tail regions of ER and ERLs. Conserved residues (Cons) are filled in black. The kinase domain is highlighted in dark gray, and ER_CT is in red. The location of the α-Helix is superimposed. Deletions used in this study are indicated in blue: ER_ΔCTα-Helix_, ER_ΔC43_, ER_ΔC56_, and ER_ΔC62_. (*B*) Structural modeling of the cytoplasmic domain of ER (ER_CD), with the juxtamembrane domain in sand, the kinase domain in gray, and the C-terminal tail in red. (*C*) In vitro phosphorylation assay of ER_CD. *Left* panels, control ER_CD and kinase-dead version (ER_CD_K676E_). *Right* panels, ER_CD and ER_CD_ΔC43_. *Top* panels, autoradiography. *Middle* panels, mean and S.D. of densitometry plotted for the experiments performed three times. * *t* test *P* < 0.05. *Bottom* panels, Coomassie Brilliant Blue staining (CBB) as a loading control.

To understand the mode of ER activation, we first compared the amino acid sequences of fourteen ER orthologs from liverworts, moss, monocots, and dicots including Arabidopsis ER paralogs, ERL1 and ERL2. In addition to the LRRs and the kinase domains, high sequence conservations were detected within the C-terminal tail regions (Fig. 1*A*). Notably, the last 15 amino acids of the C-terminal tail are nearly fully conserved with the exception of three *Physcomitrella* ERs (PpER1-3) (*SI Appendix*, Fig. S1). Next, we examined the predicted AlphaFold2 structures of the ER cytoplasmic domain (ER_CD) (46). The overall tail region does not adopt a distinct structure and flops out from the kinase domain. In contrast, the highly conserved last 15 amino acid C-terminal tail is predicted to form an α-Helix (Fig. 1*B* and *SI Appendix*, Fig. S2*A*). This predicted α-Helix exists in all the ER paralogs in Arabidopsis and orthologs from other plant species tested (e.g., *Glycine soja* and *Oryza Sativa*) (Fig. 1 and *SI Appendix*, Figs. S1 and S2*A*).

To explore whether the sequence conservation of the C-terminal tail region extends to the LRR-RK superfamily in general, we compared the tail regions of 25 representative LRR-RKs (e.g., BRI1-BRL1-BRL2-BRL3, and SERK1-SERK2-BAK1-SERK4) in Arabidopsis (*SI Appendix*, Fig. S3). The alignments suggest that the sequence similarities within the C-terminal tails are relatively limited within each receptor family. Notably, the α-Helix structure is absent in C-terminal tails of other LRR-RKs tested, including BAM1, CLV1, EFR, FLS2, HSL1, HSL2, and PSKR1 (*SI Appendix*, Fig. S2*B*). Furthermore, the tail lengths vary among these LRR-RKs, with Arabidopsis ER having the longest tail of 56 amino acids (*SI Appendix*, Fig. S3). The long C-terminal tail with the terminal α-Helix may have a specific function shared within the ER subfamily.

We next asked whether the unique C-terminal tail directly affects the kinase activity of ER (Fig. 1*B*). For this purpose, we expressed and purified an MBP-fused full-length ER_CD (MBP-ER_CD), a control kinase-inactive version in which the invariable lysine residue in the ATP binding domain is substituted to a glutamate (MBP-ER_CD_K676E_), and MBP-ER_CD_ΔC43_, in which the C-terminal 43 amino acids are removed. Subsequently, in vitro kinase assays were performed. MBP-ER_CD exhibited autophosphorylation, while MBP-ER_CD_K676E_ showed no autophosphorylation (Fig. 1*C*). Notably, MBP-ER_CD_ΔC43_ showed slight yet reproducible increase of autophosphorylation (Fig. 1*C*). To further address whether the removal of ER_CT enhances the transphosphorylation, we subjected BAK1_CD_dead_, a kinase-dead version (K364M) of BAK1 cytoplasmic domain, as a substrate for in vitro kinase assays. Indeed, the phosphorylation of BAK1_CD_dead_ was stronger after incubation with MBP-ER_CD_ΔC43_ than MBP-ER_CD (*SI Appendix*, Fig. S1*C*). Together, these results suggest that the C-terminal tail negatively regulates ER kinase activity.

### The C-Terminal Tail Negatively Regulates ER Function.

ER_CT was proposed to be dispensable for the ER function (28). However, the increased kinase activity in the absence of ER_CT suggests its potential regulatory function (Fig. 1*C*). To test this hypothesis, we first introduced three truncated versions of *ER* driven by its own promoter into *er* null allele, *er-105*: *ER_ΔCTα-Helix_, ER_ΔC56_*, and *ER_ΔC62_* lacking the C-terminal α-helix, the entire tail, and the tail plus the six last amino acids within the kinase domain, respectively (Fig. 1*A*). *ER_ΔCTα-Helix_* and *ER_ΔC56_* not only rescued *er* inflorescence phenotype but also increased their heights to approximately ~120% of control wild-type (WT) plants (*SI Appendix*, Fig. S4 *A* and *B*), suggesting that removal of the C-terminal tail confers hyperactivity of ER. On the other hand, *ER_ΔC62_* failed to rescue the compact *er* inflorescence phenotype (*SI Appendix*, Fig. S4 *A* and *B*), consistent with the importance of ER kinase activity for its function (33).

**Fig. 2.**
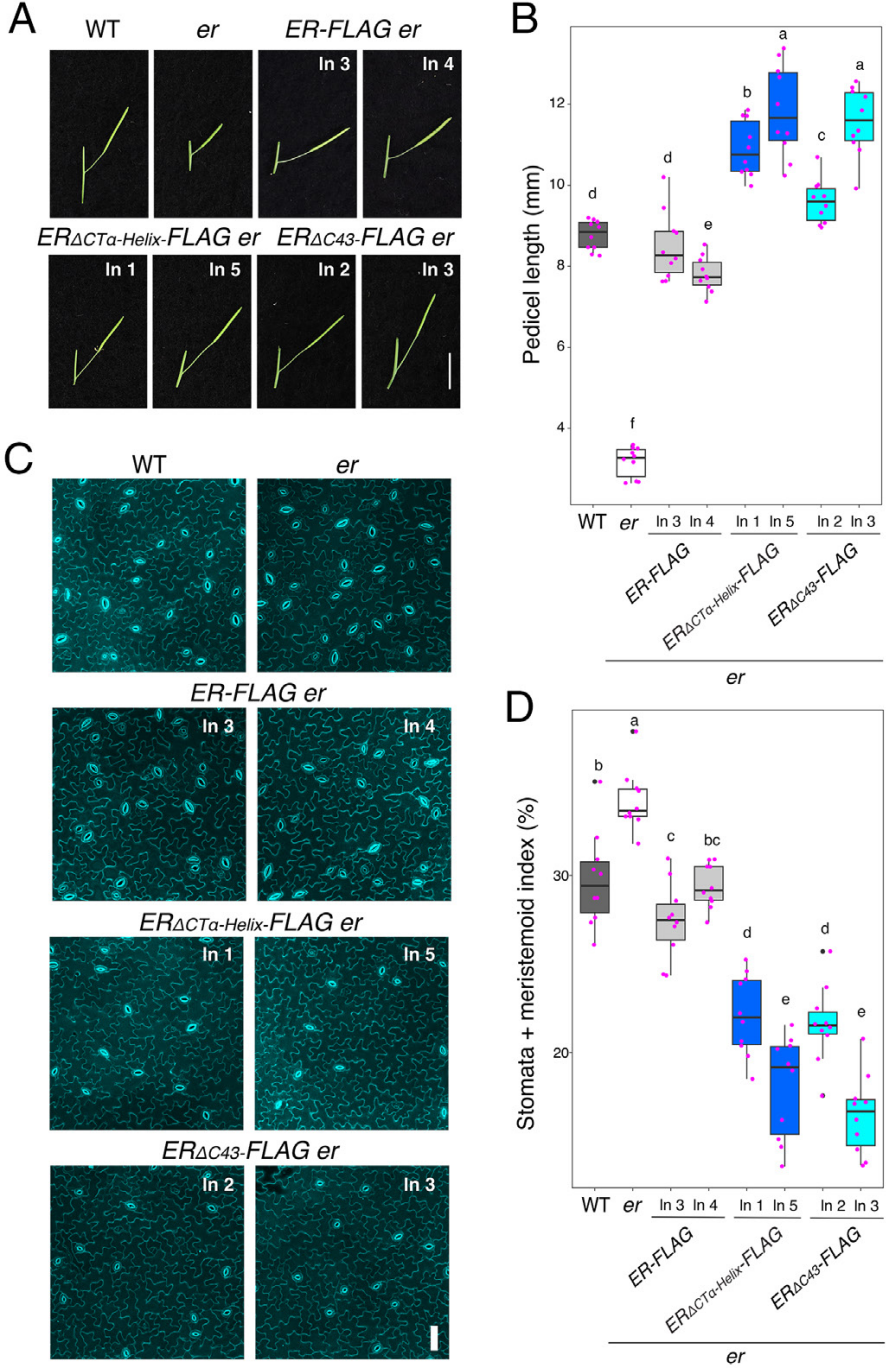
Removal of the ER C-terminal tail leads to hyperactivity in promoting pedicel elongation and inhibiting stomatal development. (*A*) Representative pedicels and mature siliques of WT, *er-105 (er)*, *ER-FLAG er*, *ER_ΔCTα-Helix_-FLAG er*, and *ER_ΔC43_-FLAG er* plants. For each transgenic construct, two representative lines were subjected to analysis. Images were taken under the same magnification. (Scale bar, 1 cm.) (*B*) Morphometric analysis of pedicel length from each genotype. 6-wk-old mature pedicels (n = 10) were measured. One-way ANOVA followed by Tukey’s HSD test was performed and classified their phenotypes into categories (e.g., *a*, *b*, *c*). (*C*) Representative confocal microscopy of cotyledon abaxial epidermal at 7 d postgermination (DPG7) from wild type (WT), *er-105* (*er*), *ER-FLAG er*, *ER_ΔCTα-Helix_-FLAG er*, and *ER_ΔC43_-FLAG er.* The same representative transgenic lines were observed. Images were taken under the same magnification. (Scale bar, 50 µm.) (*D*) Quantitative analysis. Stomata + meristemoid index (number of stomata and meristemoid per 100 epidermal cells) of the cotyledon abaxial epidermis from 7-d-old seedlings of respective genotypes (n = 10). One-way ANOVA followed by Tukey’s HSD test was performed and classified their phenotypes into categories (e.g., *a*, *b*, *c*).

We next examined the effects of these ER_CT deletions on stomatal development (Fig. 2). In *er*, clusters of small stomatal-lineage ground cells are produced due to excessive entry divisions of stomatal-lineage cells (35) (*SI Appendix*, Fig. S4*C*). The stomatal densities (number of stomata/unit area) of *ER_ΔCTα-Helix_* and *ER_ΔC56_,* but not *ER_ΔC62_*, are lower than WT (*SI Appendix*, Fig. S4*D*), suggesting that removal of ER_CT overly represses stomatal differentiation. It is known that the ER signaling pathway leads to the downregulation of the transcription factor SPEECHLESS (SPCH), which initiates stomatal cell lineages (47, 48). To further characterize the effects of *ER_ΔC_* on stomatal development at a molecular level, we further analyzed the expression levels of *SPCH* and known direct SPCH targets, *EPF2*, *TMM*, and *POLAR* (49, 50) (*SI Appendix*, Fig. S4 *E*–*H*). Both *ER_ΔCTα-Helix_* and *ER_ΔC56_* reduced the transcript levels of these genes, some with statistical significance. Thus, removal of ER_CT, either the entire domain or the α-Helix alone, is sufficient to confer hyperactivity of ER in stomatal development.

Now we know that ER_CT negatively regulates the biological function of ER, we subsequently generated *er* plants expressing the epitope-tagged versions of the truncated ER_CT for further biochemical analyses. Specifically, we focused on the deletions of conserved α-Helix domain, *ERpro::ER_ΔCTα-Helix_-FLAG,* and that enhances the in vitro kinase activity, *ERpro::ER*_Δ_*_C43_-FLAG*. Again, both *ERpro::ER_ΔCTα-Helix_-FLAG* and *ERpro::ER*_Δ_*_C43_-FLAG* conferred overcomplementation with excessive pedicel elongation (Fig. 2 *A* and *B*) and reduced numbers of stomatal and meristemoid (stomata + meristemoid index = (number of stomata and meristemoid)/number of stomata + meristemoid + all other epidermal cells) x100) compared to the control *ER-FLAG* lines (Fig. 2 *C* and *D*). The transcript levels of the transgenes are comparable among the transgenic lines expressing *ER-FLAG*, *ER_ΔCTα-Helix_-FLAG,* and *ER_ΔC43_-FLAG* (*SI Appendix*, Fig. S5*A*), indicating that the observed excessive phenotypic rescues are not attributable to the transgene overexpression. Notably, the protein levels of ER deletion versions are higher than the WT version, indicating that the C-terminal tail regulates ER protein level (*SI Appendix*, Fig. S5*B*). Based on these findings, we conclude that the ER_CT prevents the excessive activation of ER signaling and that C-terminally truncated versions of ER, whether with or without the epitope tag, exhibit hyperactivity across multiple contexts of ER-mediated developmental processes.

### The C-Terminal Tail Recruits the Inhibitory Protein of ER.

**Fig. 3.**
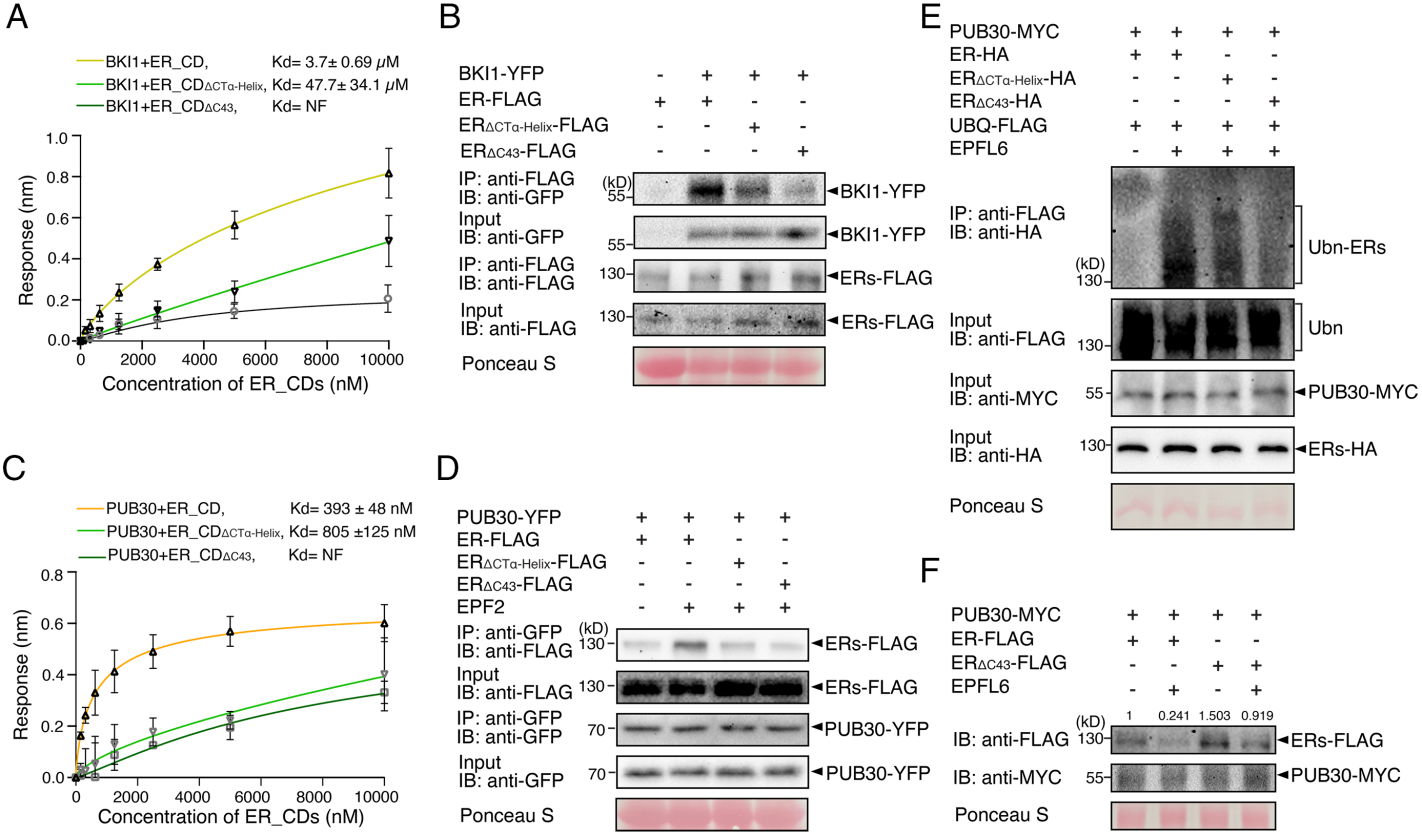
The C-terminal tail is essential for the binding of ER with BKI1 and PUB30. (*A*) Quantitative analysis of interactions between BKI1 and ER_CD variants (ER_CD, ER_CD_ΔCTα-Helix_, and ER_CD_Δ43_) using BLI. In vitro binding response curves for recombinantly purified GST-BKI1 and MBP-ER_CD variants at seven concentrations (156.25, 312.5, 625, 1,250, 2,500, 5,000, and 10,000 nM) are shown. Kd values are indicated. Data are representative of three independent experiments. (*B*) Deletion of the C-terminal tail decreases the association of ER with BKI1 in vivo. Proteins from double transgenic lines carrying *ERpro::BKI1-YFP ERpro::ER-FLAG*, *ERpro::BKI1-YFP ERpro::ER_ΔCTα-Helix_-FLAG*, and *ERpro::BKI1-YFP ERpro::ER_ΔC43_-FLAG* were immunoprecipitated with anti-FLAG beads (IP). The immunoblots (IB) were probed with anti-FLAG and anti-GFP antibodies, respectively. (*C*) Quantitative analysis of interactions between PUB30 and ER_CD variants (ER_CD, ER_CD_ΔCTα-Helix,_ and ER_CD_ΔC43_) using BLI. In vitro binding response curves for recombinantly purified GST-PUB30 and MBP-ER_CD variants at seven concentrations (156.25, 312.5, 625, 1,250, 2,500, 5,000, and 10,000 nM) are shown. Kd values are indicated. Data are representative of three independent experiments. (*D*) Deletion of the C-terminal tail decreases the association of ER with PUB30 in vivo. Proteins from *PUB30pro::PUB30-YFP; ERpro::ER-FLAG*, *PUB30pro::PUB30-YFP; ERpro:: ER_ΔCTα-Helix_-FLAG*, and *PUB30pro::PUB30-YFP; ERpro:: ER_ΔC43_-FLAG* plants were immunoprecipitated with anti-GFP beads (IP), and the immunoblots (IB) were probed with anti-GFP and anti-FLAG antibodies, respectively. (*E*) Deletion of the C-terminal tail decreases the ubiquitination of ER by PUB30 in vivo. Arabidopsis protoplasts were cotransfected with PUB30-MYC, FLAG-UBQ, together with ER-HA, ER_ΔCTα-Helix_-HA, and ER_ΔC43_-HA. Five micromolar EPFL6 was used for treatment for 1 h. After immunoprecipitation using anti-FLAG beads, the ubiquitinated ER variants were probed with anti-HA antibody. The total ubiquitinated proteins were probed by anti-FLAG antibody and PUB30 proteins were probed by anti-MYC antibody. The inputs of ER were probed with anti-HA antibody. (*F*) Representative EPFL6 treatment destabilizes ER variants in Arabidopsis protoplasts coexpressing PUB30-MYC. Protoplasts expressing the indicated proteins were treated with 50 μM CHX and 5 μM EPFL6 for 3 h. before the total protein was examined with immunoblot. The experiment was repeated independently three times with similar results. The quantification of average ER protein abundance (ERs-FLAG/Rubisco) among these three repeats was labeled.

Several possible mechanisms may explain the negative regulation of ER function by its C-terminal tail. It has been reported that BRI1 KINASE INHIBITOR1 (BKI1) inhibits ER kinase activity and regulates plant architecture together with ER (51). Therefore, we asked whether ER_CT mediates the interaction between BKI1 and ER. We first quantitatively characterized the kinetics of protein–protein interactions between BKI1 and ER_CD variants via biolayer interferometry (BLI) (*SI Appendix*, *Materials and Methods*). The BLI assay showed that BKI1 binds with ER_CD with equilibrium dissociation constant (Kd) values at 3.7 ± 0.7 μM (Fig. 3*A*). The interaction of BKI1 with ER_CD_ΔCTα-Helix_ was reduced roughly by 10-fold (Kd = 47.7 ± 34.1 μM), and no interaction was detected for BKI1 and ER_CD_ΔC43_ (Fig. 3*A*). These results suggest that the ER_CT is necessary for the association with BKI1.

Next, to examine the in vivo association of BKI1 and ER variants in Arabidopsis, we performed coimmunoprecipitation (Co-IP) analyses using transgenic plants carrying epitope-tagged ER variants (*ERpro::ER-FLAG*, *ERpro::ER_ΔCTα-Helix_-FLAG*, and *ERpro::ER*_Δ_*_C43_-FLAG*) and BKI1 (*ERpro::BKI1-YFP*). Much less BKI1-YFP signal was detected in the immunoprecipitated ER_ΔCTα-Helix_-FLAG or ER_ΔC43_-FLAG than ER-FLAG complexes (Fig. 3*B*), indicating that ER_CT is required for the in vivo binding of ER with BKI1. It has been reported that EPF/EPFL perception by ER triggers the formation and activation of the receptor complex (44, 45). To test a hypothesis that receptor activation inhibits the interaction of BKI1 and ER, we further treated the seedlings with bioactive, mature recombinant EPF2 (MEPF2) peptides. Indeed, association of ER with BKI1 decreased upon MEPF2 application (*SI Appendix*, Fig. S6). Combined, our results demonstrate that the C-terminal tail of ER is required for its interaction with BKI1 and that the ER signaling activation triggers the dissociation of BKI1.

### The C-Terminal Tail Negatively Regulates ER Protein Abundance.

A previous work illustrated that PUB30 and PUB31 down-regulate the accumulation of ligand-stimulated ER proteins via ubiquitination (45). We then asked whether ER_CT is required to recruit these E3 ligases. We found that ER_CD tightly bound with PUB30 with Kd values at 393 ± 48 nM in the BLI assays. Removal of the α-Helix reduced the association with PUB30, increasing the Kd value by a factor of two (805 ± 125 nM). Notably, ER_CD_ΔC43_ did not show any detectable binding with PUB30 (Fig. 3*C*). Like PUB30, PUB31 also exhibits weaker interaction with the ER_CD C-terminal deletion version compared with ER_CD (*SI Appendix*, Fig. S7*A*).

Next, to test their in vivo protein–protein interactions, we performed Co-IP analyses using transgenic plants expressing FLAG-tagged ER_CD variants and PUB30 (*PUB30pro::PUB30-YFP*), as well as protoplasts coexpressing epitope-tagged ER variants (ER-HA, ER_ΔCTα-Helix_-HA, and ER_∆C43_-HA) and PUB31 (PUB31-MYC) (*SI Appendix*, *Materials and Methods*). Compared to the ER, much less amounts of ER_ΔCTα-Helix_ or ER_∆C43_ proteins were detected when subjected to Co-IP with PUB30-YFP or PUB31-MYC (Fig. 3*D* and *SI Appendix*, Fig. S7*B*), indicating that, in addition to the binding with BKI1, the ER_CT is also required for the binding with PUB30/31.

To address whether ER_CT is required for the turnover of ligand-activated ER, we subsequently performed an in vivo ubiquitination assay using Arabidopsis protoplasts coexpressing epitope-tagged ER (ER-HA), PUB30 or PUB31 (PUB30-MYC or PUB31-MYC), and ubiquitin (FLAG-UBQ) (*SI Appendix*, *Materials and Methods*). Laddering bands with high molecular mass proteins are detected after immunoprecipitation, indicative of the ubiquitination of ER in vivo (Fig. 3*E* and *SI Appendix*, Fig. S7*C*). Strikingly, the deletion of ER_CT, most evident in ER_∆C43,_ diminished the polyubiquitination of ER by PUB30 and PUB31 (Fig. 3*E* and *SI Appendix*, Fig. S7*C*), suggesting that ER_CT is required for the ligand-stimulated ER ubiquitination. Finally, we tested the effects of ER_CT on the degradation of ER protein. For this purpose, we coexpressed FLAG-tagged ER variants and PUB30 in Arabidopsis protoplasts and performed cotreatment with bioactive EPFL6 peptide (MEPFL6) in the presence of cycloheximide (de novo protein synthesis inhibitor). MEPFL6 treatment substantially decreased the accumulation of ER protein but not as much of the ER_∆C43_ protein (Fig. 3*F* and *SI Appendix*, Fig. S7*B*). Based on these findings, we conclude that ER_CT is required for the interaction with PUB30/31, the ubiquitination by PUB30/31, and the turnover of ligand-stimulated ER protein.

### The ER C-Terminal Tail Is an In Vivo Phosphodomain.

In animal receptor kinases, phosphorylation of the C-terminal tail plays a role in signal transduction (52). In vivo and in vitro phosphorylation of the C-terminal tail has been reported for BRI1 and BAK1 (14, 21, 23, 53, 54). The long, conserved C-terminal tail region of ER orthologs possesses multiple serine and threonine residues that could serve as phospho-acceptor residues, many of which are highly conserved (*SI Appendix*, Fig. S1). In contrast, neither BRI1 nor BAK1 has such extended serine/threonine-rich tails (*SI Appendix*, Fig. S3). This leads us to investigate whether the ER_CT is subjected to phosphorylation and, if so, what their functional significance is.

**Fig. 4.**
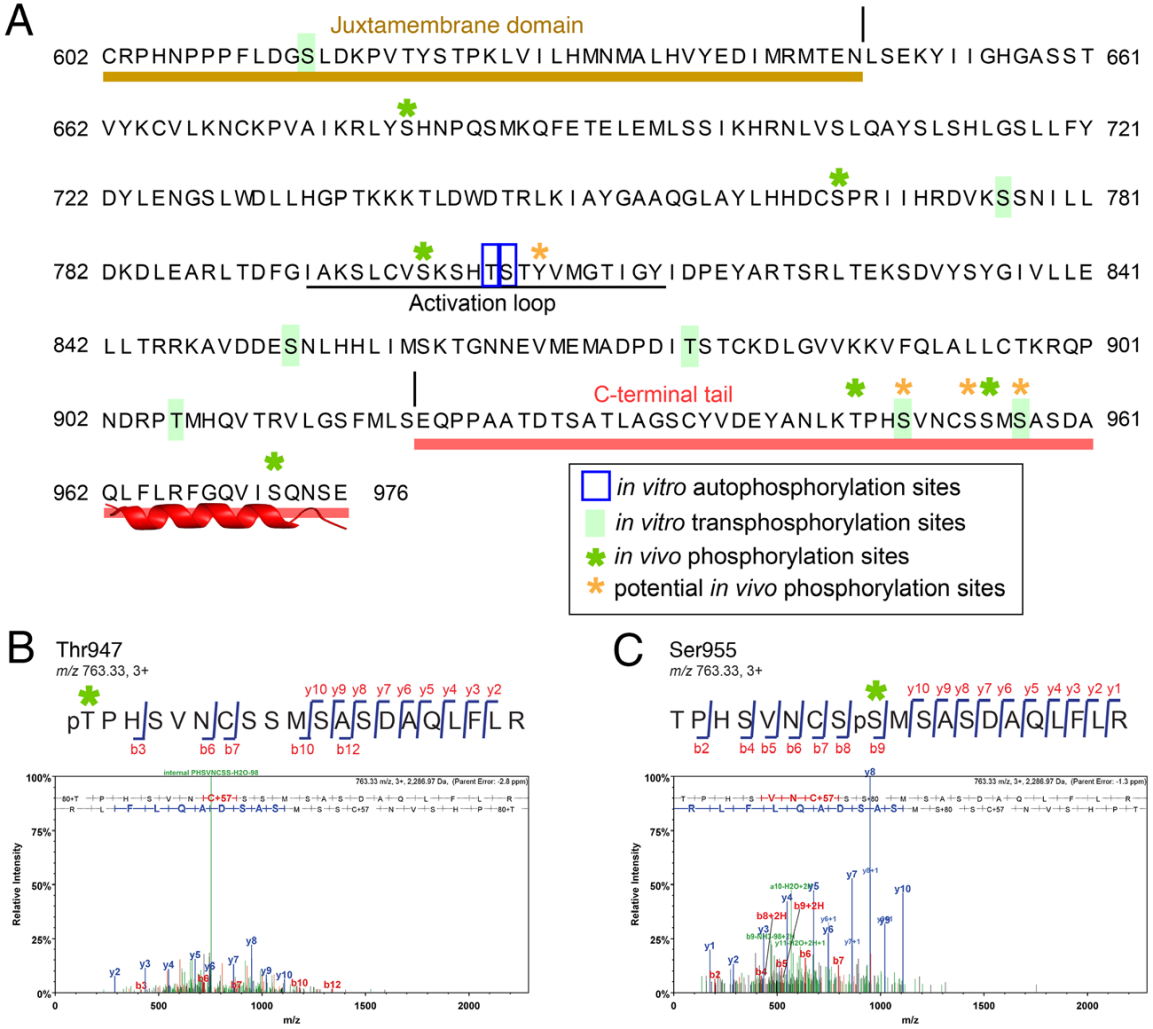
The C-terminal tail of ER is phosphorylated. (*A*) Phosphorylation sites in the C-terminal tail of ER. In vitro auto- and transphosphorylation sites identified by LC–MS/MS analysis are marked in blue and lime green, respectively. In vivo localized phosphorylation sites of ER found in this study are marked with green asterisks. Potential in vivo phosphorylation sites of ER are marked with orange asterisks. Juxtamembrane and C-terminal tail are highlighted in sand and red, respectively. Underline, the Activation Loop. (*B* and *C*) MS/MS spectra for selected in vivo phosphorylation sites of ER: Thr947 (B) and Ser955 (*C*).

To identify in vivo ER phosphosites, we performed affinity purification of Arabidopsis ER protein using transgenic Arabidopsis *er* seedlings fully complemented by functional *ER* fused with YFP driven by the endogenous promoter (*ERpro::ER-YFP*). Immunoprecipitated Arabidopsis ER-YFP protein was subjected to LC-MS/MS analysis. Eighteen experiments were performed using three different mass spectrometry experimental settings (*SI Appendix*, *Fig. S8 and Materials and Methods* for total coverage). Phosphosites Thr947, Ser955, and Ser972, along with additional phosphosites—Ser950, Ser954, and Ser957—localized with lower confidence, were detected (Fig. 4 and *SI Appendix*, Fig. S9 and Table S1). All residues are removed in ER_∆C43_. Among them, Ser955, Ser957, and Ser972 are highly conserved among the ER orthologs and paralogs (*SI Appendix*, Fig. S1), and Thr947 and Ser950 share conservation among the ER orthologs in Angiosperms and *Brassicaceae*, respectively (*SI Appendix*, Fig. S1). Thus, the highly conserved ER_CT defines an in vivo phosphodomain. Additional phosphosites were identified within the activation loop of the ER kinase domain, Ser801 and Tyr808, which are indispensable for the ER function (28), as well as Ser680 and Ser766. The recoveries of these phosphorylated peptides were low, however, implying that the ER kinase domain itself may not be predominantly phosphorylated in vivo.

### The ER C-Terminal Tail Is Transphosphorylated by BAK1.

Our work revealed that removal of ER_CT confers hyperactivity of ER (Figs. 1–3). Furthermore, we identified multiple in vivo phosphosites on the C-terminal tail (Fig. 4 and *SI Appendix*, Table S1). To delve into the underlying mechanism of phosphoregulation, we first analyzed ER autophosphorylation using MBP-ER_CD (Fig. 1*C*) (55) by mass spectrometry analysis. In contrast to the in vivo data, in vitro phosphorylation was detected only within the ER activation loop (Thr805, Ser806; Fig. 4*A* and *SI Appendix*, Table S1). As expected, no phosphopeptides were detected in the control kinase-dead ER (MBP-ER_CD_K676E_, *SI Appendix*, Table S1).

Although it has been shown by autoradiography that BAK1 can transphosphorylate ER (44), the exact phosphosites are unknown. A mass spectrometry analysis after coincubation of MBP-ER_CD with a recombinant BAK1 cytoplasmic domain (MBP-BAK1_CD), identified unambiguous phosphorylation sites at Ser950 and Ser957, and low localization score at Ser972 within the ER_CT (Fig. 4 and *SI Appendix*, Fig. S9 and Table S1). The high-confidence C-terminal tail phosphorylation occurred even when the kinase-dead ER protein (MBP-ER_CD_K676E_) was coincubated with BAK1, thereby demonstrating that the ER_CT is transphosphorylated by BAK1. Importantly, all of these in vitro transphosphorylation sites correspond to the in vivo ER phosphorylation sites (Fig. 4 and *SI Appendix*, Table S1), suggesting that these phosphosites are most likely transphosphorylated by BAK1 upon receptor complex formation in plants. In addition to the ER phosphosites, we also detected strong autophosphorylation of BAK1 in vitro (*SI Appendix*, Table S2) within the activation loop, which is required for kinase activation (24), as well as in the juxtamembrane and C-terminal tail domains (*SI Appendix*, Table S2). Importantly, in vitro BAK1 phosphosites detected in our study include all of the previously reported in vivo BAK1 phosphosites within the kinase domain (*SI Appendix*, Table S2) (14, 21, 23, 24).

### Phosphorylation of the C-Terminal Tail as a Switch for the Binding from BKI1 to PUB30/31.

**Fig. 5.**
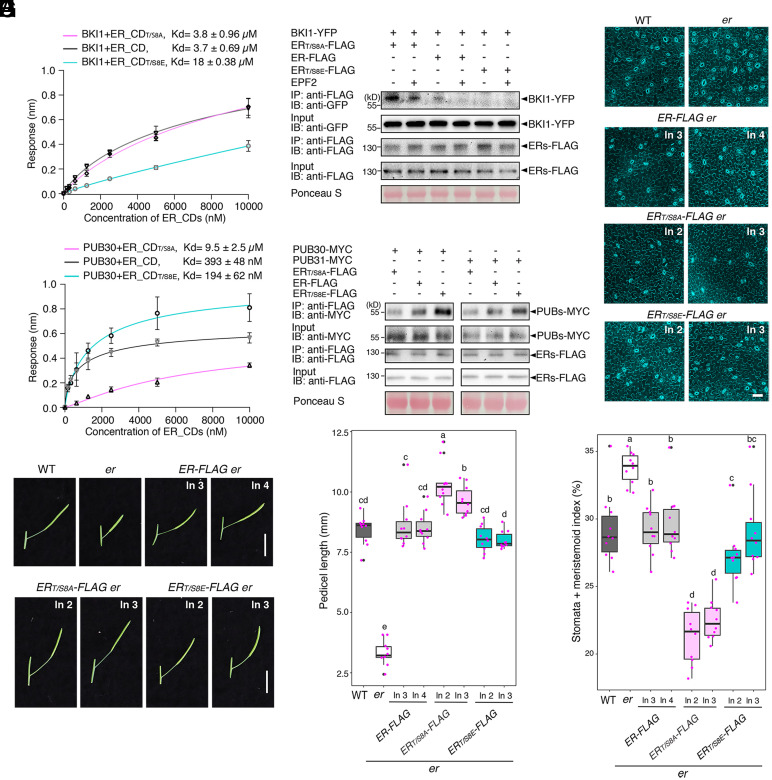
Phosphorylation of the ER C-terminal tail evicts BKI1 and recruits PUB30/31. (*A*) Quantitative analysis of interactions between BKI1 and ER_CD variants (ER_CD, ER_CD_T/S8A_, and ER_CD_T/S8E_) using BLI. In vitro binding response curves for recombinantly purified GST-BKI1 and MBP-ER_CD variants at seven concentrations (156.25, 312.5, 625, 1,250, 2,500, 5,000, and 10,000 nM) are shown. Kd values are indicated. Data are representative of three independent experiments. (*B*) Phosphorylation of the C-terminal tail decreases the association of ER with BKI1 in vivo. Proteins from *ERpro::BKI1-YFP; Col-0, ERpro::BKI1-YFP; ERpro::ER-FLAG*, *ERpro::BKI1-YFP; ERpro::ER_T/S8A_-FLAG*, and *ERpro::BKI1-YFP; ERpro::ER_T/S8E_-FLAG* plants were immunoprecipitated with anti-FLAG beads (IP), and the immunoblots (IB) were probed with anti-FLAG and anti-GFP antibodies, respectively. (*C*) Quantitative analysis of interactions between PUB30 and ER_CD variants (ER_CD, ER_CD_T/S8A_, and ER_CD_T/S8E_) using BLI. In vitro binding response curves for recombinantly purified GST-PUB30 and MBP-ER_CD variants at seven concentrations (156.25, 312.5, 625, 1,250, 2,500, 5,000, and 10,000 nM) are shown. Kd values are indicated. Data are representative of three independent experiments. (*D*) Phosphorylation of the C-terminal tail promotes the association of ER with PUB30 and PUB31 in vivo. Arabidopsis protoplasts were cotransfected with PUB30-MYC or PUB31-MYC, together with ER-FLAG, ER_T/S8A_-FLAG, and ER_T/S8E_-FLAG. Five micromolar EPFL6 was used for treatment for 1 h. After immunoprecipitation using anti-FLAG beads, the immunoblots (IB) were probed with anti-FLAG and anti-MYC antibodies, respectively. (*E*) Representative pedicels and mature siliques of WT, *er*, *ER-FLAG er*, *ER_T/S8A_-FLAG er*, and *ER_T/S8E_-FLAG er* plants. Images were taken under the same magnification. (Scale bar, 1 cm.) (*F*) Morphometric analysis of pedicel length from each genotype. 6-wk-old mature pedicels (n = 10) were measured. One-way ANOVA followed by Tukey’s HSD test was performed and classified their phenotypes into categories (*a*, *b*, *c*, and *d*). (*G*) Confocal microscopy of 7-d-old abaxial cotyledon epidermis of WT, *er*, *ER-FLAG er*, *ER_T/S8A_-FLAG er*, and *ER_T/S8E_-FLAG er* plants. Images were taken under the same magnification. (Scale bar, 50 μm.) (*H*) Quantitative analysis. Stomata + meristemoid index of the cotyledon abaxial epidermis from 7-d-old seedlings of respective genotypes (n = 10). One-way ANOVA followed by Tukey’s HSD test was performed and classified their phenotypes into categories (*a*, *b*, *c*, and *d*).

We found that ER_CT negatively regulates the kinase activity and protein abundance of ER through recruiting BKI1 and PUB30/31 before and after the activation of ER, respectively (Figs. 1 and 3). To address whether the phosphorylation status of the C-terminal tail affects the interaction of ER with BKI1 and PUB30/31, we performed site-directed mutagenesis of the eight phosphosites within ER_CT, including six experimentally detected sites and two potential sites (Ser959 and Ser975), for a series of in vitro and in vivo assays. The in vitro BLI assays showed that the phosphomimetic version ER_CD_T/S8E_ exhibits weaker interaction with BKI1 than the WT ER_CD or the phosphonull version ER_CD_T/S8A_ (Fig. 5*A*). Next, we performed in vivo Co-IP experiments using transgenic plants carrying epitope tagged-ER, *ERpro::ER-FLAG,* phosphonull version *ERpro::ER_T/S8A_-FLAG*, and phosphomimetic version *ERpro::ER_T/S8E_-FLAG* with BKI1 (*ERpro::BKI1-YFP*). The association of BKI1-YFP with ER_T/S8E_-FLAG was markedly reduced compared with the WT version (Fig. 5*B*). By contrast, the phosphonull mutant ER_T/S8A_-FLAG exhibited stronger interaction with BKI1 than the WT ER-FLAG (Fig. 5*B*). These results suggest that phosphorylation within ER_CT abolishes the binding of BKI1 and ER. Intriguingly, the phosphonull version, ER_CD_T/S8A_, exhibits much weaker interaction with PUB30 than the WT version ER_CD or the phosphomimetic version ER_CD_T/S8E_ (Fig. 5*C*). Further in vivo Co-IP assays using Arabidopsis protoplasts demonstrated both PUB30 and PUB31 show weak interaction with the phosphonull version of ER (ER_T/S8A_-FLAG) (Fig. 5*D*). In contrast, PUB30 and PUB31 exhibit stronger interaction with the phosphomimetic version of ER (ER_T/S8E_-FLAG) than the WT ER-FLAG, thereby confirming that phosphorylation of the C-terminal tail intensifies the association with its E3 ligases (Fig. 5*D*). Collectively, these findings suggest that the phosphorylation of the ER C-terminal tail leads to the eviction of BKI1 and recruitment of PUB30/31.

### Phosphorylation of the C-Terminal Tail Activates and Attenuates the ER Signaling.

To further evaluate the contribution of the C-terminal tail phosphorylation on the biological functions of ER, we introduced the C-terminal tail phosphomimetic and phosphonull versions of ER driven by its native promoter into *er* null mutant plants. Transgenic *er* plants expressing the phosphomimetic version *ERpro::ER_T/S8E_-FLAG* rescued both pedicel growth and stomatal phenotype of *er* in a similar degree to the complementation lines expressing the WT version *ERpro::ER-FLAG* (Fig. 5 *E* and *F*). Remarkably, transgenic plants expressing the phosphonull version *ERpro::ER_T/S8A_-FLAG* overly rescued the *er* mutant phenotypes, both in the context of pedicel growth and stomatal development (Fig. 5 *E* and *F*). Thus, having a C-terminal tail that cannot be phosphorylated mimics the hyperactivity of ER lacking the C-terminal tail (e.g., ER_ΔCTα-Helix_ and ER_ΔC43_) (Figs. 1 and 2 and *SI Appendix*, Fig. S4). To exclude the possibility that the various degrees of transcript levels lead to the differences in phenotypic rescues by the phosphonull *ER_T/S8A_*, we examined both transcriptional and protein levels in these transgenic lines. The transcriptional levels of phosphomimetic, WT, and phosphonull versions of *ER* are comparable (*SI Appendix*, Fig. S10*A*). Notably, the protein level of phosphonull version is higher than the WT version, while that of the phosphomimetic version is lower (*SI Appendix*, Fig. S10*B*). To confirm the effects of phosphorylation in ER_CT on the stability of ER protein, we coexpressed FLAG-tagged ER phosphovariants and PUB30 in Arabidopsis protoplasts and performed cotreatment with bioactive EPFL6 peptide in the presence of cycloheximide. MEPFL6 application substantially decreased the accumulation of WT ER protein but not as much of the ER_T/S8A_ protein (*SI Appendix*, Fig. S11*A*). Conversely, the level of ER_T/S8E_ protein was markedly lower compared to the WT ER protein, in both the absence and presence of ligand perception (*SI Appendix*, Fig. S11*B*). Taken together, our results highlight that phosphorylation of ER_CT underscores the biological functions of ER.

### Phosphorylation within the ER C-Terminal Tail Impacts Its Structure.

**Fig. 6.**
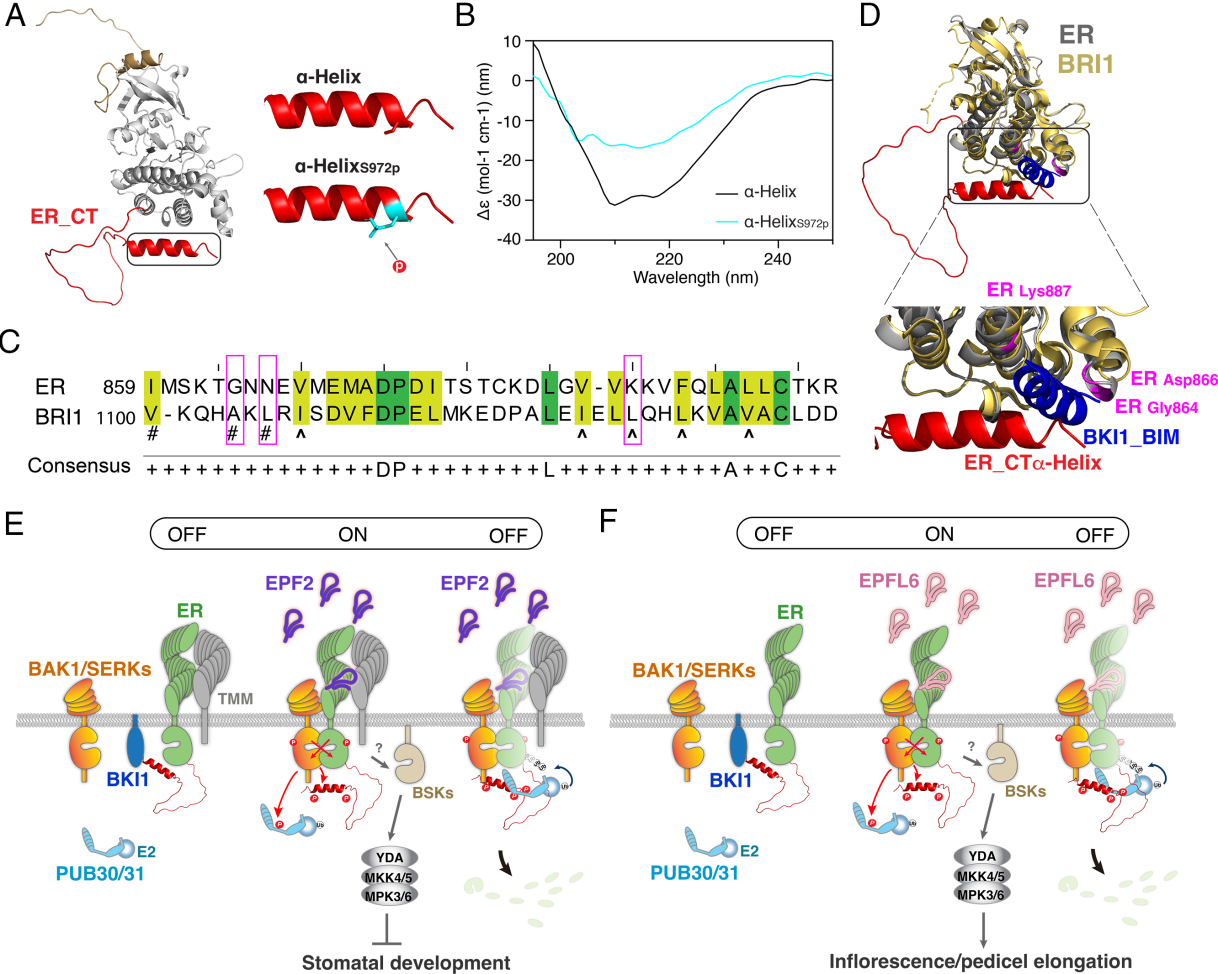
Mechanism preventing inappropriate signals pre- and postactivation of ER. (*A*) Structural modeling of ER_CD (*Left*). Structural modeling of ER_CTα-Helix domain (*Right*, *Top*), with the Ser972 residue shown as sticks in red, and ER_CTα-Helix_S972p_ (*Right*, *Bottom*), with the phosphorylated Ser972 residue shown as sticks in cyan. (*B*) CD spectra of ER_CTα-Helix (black) and ER_CTα-Helix_S972p_ (cyan). (*C*) Alignment of ER_KD and BRI1_KD. Based on the importance for the phosphorylation efficiencies toward BKI1, the crucial and minor residues in BRI1 are labeled with “^” and “#”, respectively. Conserved amino acids between ER_KD and BRI1_KD are highlighted in green for identical residues and yellow for residues with similar properties. Nonconserved amino acids are marked with magenta brackets. (*D*) Structural superimposition of the BRI1–BIM complex (PDB, 4OH4) and the ER AlphaFold2 model, highlighting BKI1_BIM in blue, BRI1_KD in yellow orange, ER_KD in gray, ER_CT in red, and residues in ER that are not conserved in magenta. (*E* and *F*) Regulation of stomatal development (*E*) and inflorescence/pedicel elongation (*F*) by ER_CT. (*Left*, the 1st state, OFF) BKI1 (blue) associates with ER (green) in the absence of ligand and ER is in the basal state. (*Middle*, the 2nd state, ON) Upon perception of EPF2 (*E*, violet) or EPFL6 (*F*, pink), ER signaling becomes activated. Both the ER kinase domain and C-terminal tail are phosphorylated by its coreceptor BAK1/SERKs (orange) during the transphosphorylation events. The phosphorylation of the C-terminal tail evicts BKI1. The activated ER-BAK1/SERKs receptor complex transduces signals most likely via BSKs (sand) before the activation of a MAPK cascade and subsequent inhibition of stomatal development (*E*) or promotion of inflorescence/pedicel elongation (*F*). TMM (gray) biases the signal activation for stomatal development (*E*). (*Right*, the 3rd state, OFF) The phosphorylated ER_CT recruits the E3 ligases PUB30/31 (cyan), which, after being phosphorylated by activated BAK1/SERKs, ubiquitinate ER (dimmed green) for eventual degradation to ensure the robust yet appropriate signaling activation upon ligand perception.

The AlphaFold2 prediction of ER_CT adopts a flexible structure, ending with an α-Helix (Fig. 1 and *SI Appendix*, Fig. S3). The Ser972 residue, one of the in vivo and in vitro transphosphorylation sites, is located within the ER_CTα-Helix. This serine residue is highly conserved among the ER orthologs and paralogs (*SI Appendix*, Fig. S1), and its phosphorylation is predicted to affect the flexibility of the α-Helix (Fig. 6*A*). We postulated that the phosphorylation within the ER_CT may impact its secondary structure. To explore this possibility experimentally, we employed circular dichroism (CD) spectroscopy to analyze the secondary structures of a synthetic peptide (ER_CTα-Helix) and its phosphorylated form at the Ser972 residue (ER_CTα-Helix_S972p_) (Fig. 6*B*). The CD spectra confirmed the dominance of the predicted α-Helix in the last 15 aa of the ER_CT. Further secondary structure analysis indicates that the Ser972 phosphorylation reduces the Helix content from 86.0 % to 74.5 % (Fig. 6*B*), suggesting that the phosphorylation of ER_CT impacts its structure.

Our work demonstrates that the phosphorylation of ER_CT leads to the dissociation of BKI1 (Fig. 5 *A* and *B*). We thus postulated that a structural change within ER_CTα-Helix upon phosphorylation influences the binding of ER with BKI1. A structure of the BRI1 kinase domain (BRI1_KD) complexed with the BKI1_BIM peptide shows that BIM binds to a conserved surface patch in the C-terminal lobe of BRI1_KD (15). We first performed the protein sequence alignment of the ER kinase domain (ER_KD) and BRI1_KD. As shown in Fig. 6*C*, the amino acid sequences at the BIM interaction surfaces are not highly conserved between BRI1 and ER. In detail, one crucial residue of BRI1 (BRI1 _Leu1128_) for the phosphorylation of BKI1 as well as two residues of minor importance (BRI1 _Ala1104_ and BRI1 _Leu1106_) (15) are absent in ER_KD. Thus, the mode of BKI1 binding may be different between ER and BRI1. We further performed a structural superimposition of the BRI1–BIM peptide complex (PDB: 4OH4) (15) and the ER AlphaFold2 model. Strikingly, the superimposed structure located ER_CTα-Helix at the proximity of the BIM binding region (Fig. 6*D*), possibly emphasizing its importance for the interaction with BKI1. Taken together, we propose that phosphorylation of ER_CT by BAK1, triggered by the EPF/EPFL ligand perception and subsequent ER-BAK1 heterodimerization, induces a structural change that alleviates ER autoinhibition and promotes the dissociation of BKI1 (Fig. 6 *E* and *F*).

## Discussion

Based on our findings, we propose a regulatory mechanism of the cell-surface receptor as an off–on–off toggle switch, enabling the swift transition of signaling from inhibition, activation, to attenuation in response to ligand perception (Fig. 6*C*). A series of biochemical analyses illustrate that the C-terminal tail of ER possesses a dual role in recruiting both the inhibitory protein BKI1 and the E3 ligases, PUB30/31, in the basal and activated state, respectively. The full activation of ER-BAK1 signaling via transphosphorylation of the ER C-terminal tail likely evokes a structural change within its α-Helix (Fig. 6 *A* and *B*). This event involves the relief of both intramolecular inhibition and intermolecular interactions with BKI1 (Figs. 5 and 6). Thereafter, the phosphorylation events trigger the association of ER with E3 ligases, which eventually leads to the turnover of ER (Fig. 5 and *SI Appendix*, Fig. S10). Our work sheds light not only on the mechanism of receptor autoinhibition but also that of receptor activation and attenuation orchestrated by the multilayered phosphoregulation of a receptor complex.

It is widely accepted that phosphorylation events in the C-terminal tails are highly relevant to the activity of receptor-kinase signal transduction (13, 52, 56). However, the exact modes of action appear quite distinct, even among the plant LRR-RKs. For instance, we have shown that the ER C-terminal tail can be transphosphorylated by BAK1 (*SI Appendix*, Tables S1 and S2), suggesting that it is part of a signal transduction process after BAK1 associates with ER upon ligand perception. The BAK1-mediated phosphorylation of the ER C-terminal tail further dissociates BKI1. In contrast, a well-known LRR-RK, BRI1, exhibits strong intermolecular transphosphorylation of its own C terminus in vitro, suggesting that its C-terminal tail phosphorylation signifies the initial activation step of the BRI1 homodimers (14). Subsequent BAK1-mediated BRI1 transphosphorylations then fully activate the BR signaling (21). In fact, BAK1 per se was not able to phosphorylate the BRI1 C-terminal tail, but BAK1 could enhance BRI1 kinase activity (21). It is also worth pointing out that, unlike the dispensable role of the C-terminal tail in the BRI1–BKI1 interaction (15, 56, 57), ER_CT is crucial for ER to bind with BKI1 and to eventually evict it (Figs. 3 and 6). Indeed, amino acid residues of BRI1 at the BKI1_BIM interaction interface are not well conserved in ER, further emphasizing the unique mode of action of ER_CT (Fig. 6*C*). Thus, although both BRI1 and ER share BAK1/SERKs as heterodimeric partners (18, 19, 44), the exact mechanism of receptor activation and subsequent signaling may be distinct (Fig. 6). This idea echoes a recent finding that different BAK1 phosphocodes specify BRI1 *vs*. FLS2 LRR-RK signaling (23). More recently, BAK1-mediated phosphorylation of an LRR-RK, BAK-TO-LIFE2 (BTL2), was shown to function as a phosphoswitch to prevent autoimmunity (58). This phosphorylation occurs within the juxtamembrane domain (but not the C terminus) of BTL2, therefore highlighting the diverse mechanisms of LRR-RK phosphoregulation.

Our work further revealed that the ER C-terminal α-Helix directly contributes to the toggle switch function. Indeed, the deletion of the C-terminal α-Helix alone alleviates BKI1 inhibition and diminishes signal attenuation mediated by PUB30 and PUB31 as much as the removal of the entire ER C-terminal tail, thereby resulting in stronger signal output and hyperactivity in both stomatal development and inflorescence growth (Figs. 2 and 6 and *SI Appendix*, Fig. S4). Furthermore, the structural modeling provides a possible role of ER_ CTα-Helix in regulating BKI1 association and eviction (Fig. 6*D*). Does this mean that the region outside of the α-Helix plays an insignificant role? We argue that the remaining C-terminal tail provides a critical interaction interface with the key ER signaling components. Indeed, removal of the C-terminal 43 amino acids (ER_ΔC43_) further compromised the association of ER with both BKI1 and PUB30/31 (Fig. 2). The additional ER C-terminal tail region outside of the α-Helix possesses multiple transphosphorylation sites important for the ER protein stability as well as protein–protein interactions (Figs. 4 and 5 and *SI Appendix*, Figs. S9 and S11 and Table S1). Thus, this region likely forms an unstructured flexible linker to function as a phosphorylation-regulated hinge to change binding partners (Fig. 6). A similar mechanism has been proposed for human ribosomal S6 kinase1, in which C-terminal tail phosphorylations within the disordered domain trigger both charge- and conformational changes, exposing the protein–protein interaction surfaces (59). In such a case, the phosphosites may act as an on–off switch or a dimmer to collectively attenuate the signal output. Likewise, a mode of autoinhibition via the C-terminal tail and following release via phosphorylation also exists in H^+^-ATPase during blue light activation of stomatal opening, suggesting a broad role of CTs as roadblocks and removal of roadblocks via phosphorylation in plants as well (60).

This study provides further insight into the role of C-terminal tail phosphoregulation in plant receptor kinases. The length and sequence of the C-terminal tails are highly variable among the plant LRR-RK families, with some members (e.g., PSKR1, CLV1, and BAM3) lacking or having very short C-terminal tails (*SI Appendix*, Fig. S2). It is known that ER-family LRR-RKs simultaneously perceive multiple ligands emanating from the neighboring cells and tissues, some with redundant or antagonistic functions (27, 61). The unique structure and function of the ER C-terminal tail may be important for avoiding signal interference to achieve precise information processing during diverse developmental processes mediated by ER-family RKs. Further studies in the ER-family and other RKs will elucidate the conserved and unique modes of receptor kinase activation and attenuation.

## Materials and Methods

Plant materials and growth conditions, plasmid construction and generation of transgenic plants, microscopy, quantitative analysis and statistics, expression, purification, and refolding of peptides, Co-IP, protein gel electrophoresis, and immunoblots, in vivo ubiquitination assays, in vitro kinase assays and detection of phosphosites by mass spectrometry, detection of in vivo phosphorylation sites by mass spectrometry, BLI, ER protein stability assay in protoplasts, RT-qPCR analysis, and CD spectroscopy are described in *SI Appendix*, *Materials and Methods*.

## Supplementary Material

Appendix 01 (PDF)

## Data Availability

The mass spectrometry proteomics data have been deposited to the ProteomeXchange Consortium (identifiers PXD056259 and PXD058419) (62, 63) via MassIVE partner repository with the dataset identifier MSV000095956 and PRIDE partner repository with the dataset identifier PXD058419 and 10.6019/PXD058419, respectively (62, 63). Spectrum Reports for Scaffold PTM of mass spectrometry analysis are also available at Texas Dataverse (10.18738/T8/Y5IOXM) (64).
