# Supplementary material for: Preventing inappropriate signals pre- and post-ligand perception by a toggle switch mechanism of ERECTA
Source: Proc Natl Acad Sci U S A. 2025 Jan 22;122(4):e2420196122. doi: 10.1073/pnas.2420196122 (PMC11789017; doi:10.1073/pnas.2420196122)
Supplement: Supplementary file 1 — Appendix 01 (PDF) [file pnas.2420196122.sapp.pdf]

PNAS

Supporting Information for

Preventing Inappropriate Signals Pre- and Post-Ligand Perception by a Toggle Switch Mechanism of ERECTA

Liangliang Chen, Michal Maes, Alicia M. Cochran, Julian R. Avila, Paul Derbyshire, Jan Sklenar, Kelsey M. Haas, Judit Villén, Frank L.H. Menke, and Keiko U. Torii

Keiko U. Torii
Email: ktorii@utexas.edu

This PDF file includes:

SI Materials and Methods
Figures S1 to S11
Tables S1 to S4
SI References

SI Materials and Methods

Plant materials and growth conditions

The Arabidopsis Columbia (Col) accession was used as the wild type. All plants used in this study are in Col background. The following mutants and transgenic plant lines were reported previously: *er-105*; *pub30 pub31*; *PUB30pro::PUB30-YFP* in *pub30 pub31*, and *PUB31pro::PUB31-YFP* in *pub30 pub31* (1). Arabidopsis seeds were surface sterilized with 30 % bleach and grown on ½ Murashige and Skoog (MS) media containing 1x Gamborg Vitamin (Sigma), 0.75 % Bacto Agar, and 1 % w/v sucrose for 9 days and then transplanted to soil. Plants were grown under long-day conditions (16-h-light /8-h-dark) at 22 °C.

Plasmid construction and generation of transgenic plants

For recombinant protein expression, the following plasmids were generated: pJA51 (MBP-ER_CD), pKUT537 (MBP-ER_CD$_{K676E}$), pJA69 (MBP-ER_CD$_{\Delta C43}$), pMM159 (MBP-ER_CD$_{\Delta CT\alpha\text{-}Helix}$), pCLL107 (GST-PUB30), pCLL109 (GST-PUB31), pCLL278 (GST-BKI1), pCLL276 (MBP-ER_CD$_{T/S8A}$), pCLL277 (MBP-ER_CD$_{T/S8E}$). Site-directed mutagenesis was performed using a 2-sided PCR overlap extension followed by assembly into linearized vectors pGEX-4T-1 and pMAL-C2. For complementation assays, the following plasmids were generated: pKUT603 (*ERpro::gER*$_{\Delta CT\alpha\text{-}Helix}$), pKUT604 (*ERpro::gER*$_{\Delta C56}$), pKUT605 (*ERpro::gER*$_{\Delta C62}$), pCLL202 (*ERpro::gER-FLAG*), pCLL261 (*ERpro::gER*$_{\Delta CT\alpha\text{-}Helix}$*-FLAG*), pCLL262 (*ERpro::gER*$_{\Delta C43}$*-FLAG*), pCLL272 (*ERpro::gER*$_{T/S8A}$*-FLAG*), pCLL273 (*ERpro::gER*$_{T/S8E}$*-FLAG*). A Three-way Gateway system (2) was utilized to generate a series of *ER* constructs driven by the *ER* promoter. See Tables S3 and S4 for details of plasmid and oligo DNA information. Plasmids are transformed into Agrobacterium GV3101/pMP90 and subsequently to Arabidopsis by floral dipping. Over 10 lines were characterized for the phenotypes and reporter gene expressions.

Microscopy

Confocal microscope images were taken as described previously using either Zeiss LSM700 operated by Zen2009 (Zeiss) or Leica SP5-WLL operated by LAS AF (Leica). Cell peripheries of seedlings were visualized with propidium iodide (Molecular Probes). Images were taken with excitation at 514 nm and emission at 518–600 nm for YFP, excitation at 619 nm, and emission at 642 nm for propidium iodide. For SP-5, a HyD detector was used. The confocal images were false-colored, and brightness/contrast were uniformly adjusted using Photoshop 2021 (Adobe). The Z-stack projection images were taken at the interval of 0.99 µm, covering the thickness of the entire cotyledon.

Quantitative analysis and statistics

For analysis of the epidermis, abaxial cotyledons from 7- or 10-d-old seedlings of relevant genotypes were subjected to PI staining and confocal microscopy. The central regions overlying the distal vascular loop were imaged and numbers of epidermal cells, stomata, and their cluster size were quantified. Pedicel lengths were measured using ImageJ. Statistical analysis was performed using R ver. 4.1.0 operated under R-Studio ver. 1.4.1717 (https://www.rstudio.com), and graphs were generated using R ggplot2 package. For all boxplots, each box represents IQR (interquartile range), in which the top, middle line, and bottom indicate 75, 50, and 25 percentiles, respectively, and the bar represents maximum to minimal values. Each dot represents a jittered individual data point. The data points that go beyond the bar are outliers. For all analyses, Welche's unpaired t-test or Student's t-test was performed for a pairwise comparison, and One-way ANOVA followed by Tukey's HSD test was performed for a comparison of groups. For individual sample size (n) and p values, see corresponding Figures or Figure Legends.

Expression, purification, and refolding of peptides

Recombinant MEPF2 and MEPFL6 peptides were prepared as described previously (3-5). Bioactivities of refolded peptides were confirmed as described previously (3).

Co-immunoprecipitation, protein gel electrophoresis, and immunoblots

For co-immunoprecipitation (Co-IP) assays with seedlings, we generated Arabidopsis transgenic lines expressing *PUB30pro::PUB30-YFP* or *ERpro::BKI1-YFP* with different versions of *ER* driven by its own promoter (*ERpro::ERs-FLAG)*. For peptide treatment, Arabidopsis seedlings were grown for five days on ½ MS media plates and then transferred to ddH_2O for 24 hrs. Seedlings were firstly treated with 50 μM MG132 (M7449, Sigma) for 3 hrs. Thereafter, further treatment was performed with Tris-HCl (pH8.0, 50mM) buffer only, MEPF2 (2.5 μM) at room temperature for another 3 hrs before being pooled for harvest and then subjected to protein preparation.

The samples were ground in liquid nitrogen and homogenized in the extraction buffer (50 mM Tris-HCl, pH 7.5, 150 mM NaCl, 1 mM EDTA, 20% glycerol, 1% Triton X-100, and 1x cOmplete protease inhibitor cocktail from Roche, 1x phosphatase inhibitor cocktail 2 and 3 from Sigma). The supernatant was incubated with Protein G-coupled magnetic beads (Dynabeads Protein G, Invitrogen) that captured anti-FLAG (ab205606; Abcam) antibody at 4 °C for 2 hrs with gentle agitation. Then, the beads were washed three times with 500 μl of wash buffer (50 mM Tris-HCl, pH 7.5, 150 mM NaCl, 1 mM EDTA, 20% glycerol, 0.2% Triton X-100, and 1x cOmplete protease inhibitor cocktail from Roche, 1x phosphatase inhibitor cocktail 2 and 3 from Sigma) and precipitated proteins were eluted with 4x SDS sample buffer at 95 °C for 5 minutes. Either total membrane or immunoprecipitated proteins were separated on SDS-PAGE gels and transferred to PVDF membrane (Millipore) for immunoblot analysis using monoclonal anti-GFP (33-2600, 1:1,000, Thermo Fisher Scientific), anti-FLAG (F-3165, 1:5,000, Sigma), and anti-BAK1 (AS12 1858, 1:5,000, Agrisera) as primary antibodies. As secondary antibodies, goat anti-mouse IgG H&L (HRP) (ab205719, Abcam) and goat anti-rabbit IgG H&L (HRP) (ab205718, Abcam) were used at a dilution of 1:5,000 and 1:5,000, respectively. The protein blots were visualized using a Chemi-luminescence assay kit (34095, Thermo Scientific) and Image Lab (Bio-Rad, version 6.0.1).

For Co-IP assays with Arabidopsis protoplasts, protoplasts were transfected with HA-tagged ER variants and MYC-tagged PUB30 or PUB31 and incubated for 8 hrs. Then, protoplasts were pretreated with 2 μM MG132 (M7449, Sigma) for 1 hr, followed by treatment with 5 μM EPFL6 for 1 hr. The total proteins were isolated with the extraction buffer described above. The supernatant was incubated with Protein G-coupled magnetic beads (Dynabeads Protein G, Invitrogen) that captured anti-MYC (ab9106; Abcam) antibody at 4 °C for 2 hrs with gentle agitation. Then, the beads were washed three times with 500 μl of wash buffer described above, and precipitated proteins were eluted with 4x SDS sample buffer at 95 °C for 5 minutes. Either total or immunoprecipitated proteins were separated on SDS-PAGE gels and transferred to PVDF membrane (Millipore) for immunoblot analysis using anti-HA (ab18181, 1:1,000, Abcam), and anti-MYC (ab32, 1:1,000, Abcam) as primary antibodies. As a secondary antibody, goat anti-mouse IgG H&L (HRP) (ab205719, Abcam) was used at a dilution of 1:5,000. The protein blots were visualized using a Chemi-luminescence assay kit (34095, Thermo Scientific) and Image Lab (Bio-Rad, version 6.0.1).

***In vivo* ubiquitination assays**

Arabidopsis protoplasts were co-transfected with FLAG-tagged ubiquitin (FLAG-UBQ), HA-tagged ER variants and together with a control vector or MYC-tagged PUB30 or PUB31 and incubated for 8 hrs followed by treatment with 5 μM EPFL6 for 1 hr in the presence of 2 μM MG132 (M7449, Sigma). The ubiquitinated ER was detected with α-HA (ab18181, 1:1,000, Abcam) IB after IP with α-FLAG (ab205606, Abcam) antibody. The total ubiquitinated proteins were detected by anti-FLAG (F-3165, 1:5,000, Sigma) and α-MYC (ab32, 1:1,000, Abcam) as primary antibodies. As a secondary antibody, goat anti-mouse IgG H&L (HRP) (ab205719, Abcam) was used at a dilution of 1:50,000. The protein blots were visualized using a Chemi-luminescence assay kit (34095, Thermo Scientific) and Image Lab (Bio-Rad, version 6.0.1).

***In vitro* kinase assays and detection of phosphosites by mass spectrometry**

Kinase assays were conducted using 1 μg of purified recombinant kinase MBP_ER_CD or MBP-ER_$CD_{\Delta C43}$ in 30 μl reactions containing 20 mM Tris-HCl pH7.5, 1mM DTT, 100 mM NaCl, 10 mM $MgCl_2$, 100 μM [γ-^{32}P] ATP mix (5 μCi of ATP; ATP, [γ-^{32}P]-3000 Ci $mmol^{-1}$, 10 mCi ml^{-1}; EasyTide, 100 μCi) and 10 mM $MnCl_2$. For the transphosphorylation analysis, 10 μg of substrate, BAK1_CD_{dead} protein (6), was incubated with 1 μg of kinase, MBP_ER_CD, or MBP-ER_$CD_{\Delta C43}$.

Reactions were incubated at 30 ºC for 60 minutes and stopped with SDS sample buffer. Proteins were resolved in 10% SDS-PAGE, and gels were dried and subsequently exposed to a GE Multipurpose Standard Screen (63-0034-87) and imaged using a GE Typhoon FLA 9000 Gel imager. Phosphorylation intensities were quantitated using ImageJ. Statistical analyses were done on three independent replicates.

For detection of *in vitro* phosphosites, purified *E. coli* expressed MBP-ER_CD, MBP-BAK1_CD, MBP-ER_CD$_{K676E}$, and MBP-BAK1_CD$_{dead}$ proteins were incubated with reaction buffer with cold ATP for 4 hrs and subjected to SDS-PAGE. The corresponding bands were excised, trypsin digested and analyzed by using a Q Exactive Quadrupole-Orbitrap Mass Spectrometer (ThermoFisher Scientific) as described previously (7). Search parameters were set as follows: enzyme selected with two maximum missing cleavage sites, a mass tolerance of 10 ppm for peptide tolerance, 0.02 Da for MS/MS tolerance, fixed modification of carbamidomethylation (C), variable modifications of oxidation (M) and phosphorylation (S, T, Y).

Detection of *in vivo* phosphorylation sites by mass spectrometry

ERpro::ER-YFP in *er-105* seedlings were germinated and grown in liquid ½ MS medium with 1 % sucrose for 5 days on an orbital shaker at 120 rpm, harvested and frozen in liquid N_2. Proteins were extracted by coarsely grinding tissue under liquid N_2 and homogenization in extraction buffer (50 mM Tris pH 7.5, 100 mM NaCl, 10% glycerol, 5 mM DTT, 1 mM NaF, 2 mM Na_3VO_4, 2 mM sodium β-glycerophosphate, 1 mM PMSF and Sigma protease inhibitor cocktail) in a 60-ml Potter-Elvehjem homogenizer on ice (8 min at 1000 rpm). After homogenization, IGEPAL CA-360 (Sigma) was added to a final concentration of 1%, and the homogenate was incubated at 4 °C for one hour to allow solubilization of membrane proteins. The homogenate was centrifuged for 20 min at 10,000 rpm. Immunoprecipitation of ER-YFP was performed as described previously (8) using anti-GFP µMACS magnetic beads (Miltenyi Biotec). Beads were washed twice with extraction buffer with 0.5% IGEPAL CA360 (Sigma-Aldrich). Proteins were eluted in a pre-warmed SDS-PAGE loading buffer and subjected to SDS-PAGE. SDS-PAGE gels were stained with Coomassie Brilliant Blue (Simply Blue ™ Safe stain, Invitrogen), and gel bands were excised for MS analysis. In-gel digestion with trypsin was performed exactly as described previously (8). LC-MS/MS analysis was performed using an Orbitrap Fusion tribrid mass spectrometer (Thermo Scientific) and a nanoflow-UHPLC system (Dionex Ultimate3000, Thermo Scientific) as described previously (8). Raw files were converted and searched as described previously (8) using ScaffoldPTM software (http://www.proteomesoftware.com/products/ptm/). For results shown in Table S1B, gel bands were cut in half, reduced with dithiothreitol, alkylated with iodoacetamide, and digested with either trypsin or chymotrypsin using standard protocols. Peptides were desalted over C18 stage-tips. Samples were analyzed on an LTQ-Velos Orbitrap (Thermo Scientific), collecting MS scans in the orbitrap and MS/MS scans in the ion trap. RAW files were converted to mzXML files and searched with Comet against the *Arabidopsis thaliana* protein sequence database to which we appended ER-YFP. A-Score was used for site localization, in which lower than 13 is considered ambiguous.

Biolayer interferometry (BLI)

The binding affinities of the ER_CD variants with GST-tagged BKI1, PUB30, and PUB31 were measured using the Octet Red96 system (ForteBio, Pall Life Sciences) following the manufacturer's protocols. The optical probes coated with anti-GST were first loaded with 2000 nM GST-BKI1, GST-PUB30, or GST-PUB31 before kinetic binding analyses. The experiment was performed in 96-well plates maintained at 30 °C. Each well was loaded with 200 µl reaction volume, and the binding buffer used in these experiments contained 1× PBS supplemented with 5 mM DTT. The concentrations of the ER_CD variants as the analyte in the binding buffer were 10000 nM, 5000 nM, 2500 nM, 1250 nM, 625 nM, 312.5 nM, and 156.3 nM. All preformed complexes remained stable as suggested by the constant signal during the washing step after loading. There was no binding of the analytes to the unloaded probes as shown by the control wells. Binding kinetics to all seven concentrations of the analytes were measured simultaneously using default parameters on the instrument. The data were analyzed using the Octet data analysis software. The association and dissociation curves were fit with the 1:1 homogeneous ligand model. The kobs (observed rate constant) values were used to calculate K_d, with steady-state analysis of the direct binding.

ER protein stability assay in protoplasts

To determine ER protein stability, protoplasts co-transfected PUB30/31-MYC with WT, C-terminal tail deletion, or phosphor-mutant versions ER-FLAG were treated with 50 μM cycloheximide (CHX, C4859, Sigma) in the presence or absence of 5 μM MEPFL6 for 3 hrs. Total proteins were separated on SDS-PAGE gels and transferred to the PVDF membrane (Millipore) for immunoblot analysis. ER variants protein and the input PUB30 or PUB31 proteins were detected with α-FLAG antibody (F-3165, 1:5,000, Sigma) and α-MYC antibody (ab32, 1:1,000, Abcam) as primary antibodies, respectively. As a secondary antibody, goat anti-mouse IgG H&L (HRP) (ab205719, Abcam) was used at a dilution of 1:5,000. The protein blots were visualized using a Chemi-luminescence assay kit (34095, Thermo Scientific) and Image Lab (Bio-Rad, version 6.0.1).

RT-qPCR analysis

Tissues for qPCR analysis were harvested at 3 day-post-germination for established lines or ~6 true leaves from T1 seedlings. RNA extraction, cDNA synthesis, and RT-qPCR were performed as described previously (9). Transcript levels were normalized against *ACTIN* (*ACT2*). For primer DNA sequences used for RT-qPCR analysis, see Table S4.

Circular Dichroism (CD) spectroscopy

All CD spectra were collected on a Jasco J-815 Circular Dichroism Spectrometer at 25 °C in PBS buffer, pH 7.4. Peptides of ER_CTα-Helix and Ser972 phosphorylated ER_CTα-Helix (ER_CTα-Helix$_{S972p}$) were synthesized commercially (Bio-Synthesis, Lewisville, TX) and dissolved in the PBS buffer before determination of peptide concentration. To quantify the short peptides, a tryptophan residue was added to the N-terminus of each peptide. Spectra were recorded from 250 to 195 nm with a scan rate of 20 nm/min in a 0.1 cm path length cuvette. The final spectral measurements representing an average of five independent scans were corrected for buffer contribution. The secondary structures of ER_CTα-Helix and ER_CTα-Helix$_{S972p}$ were predicted as described (10).

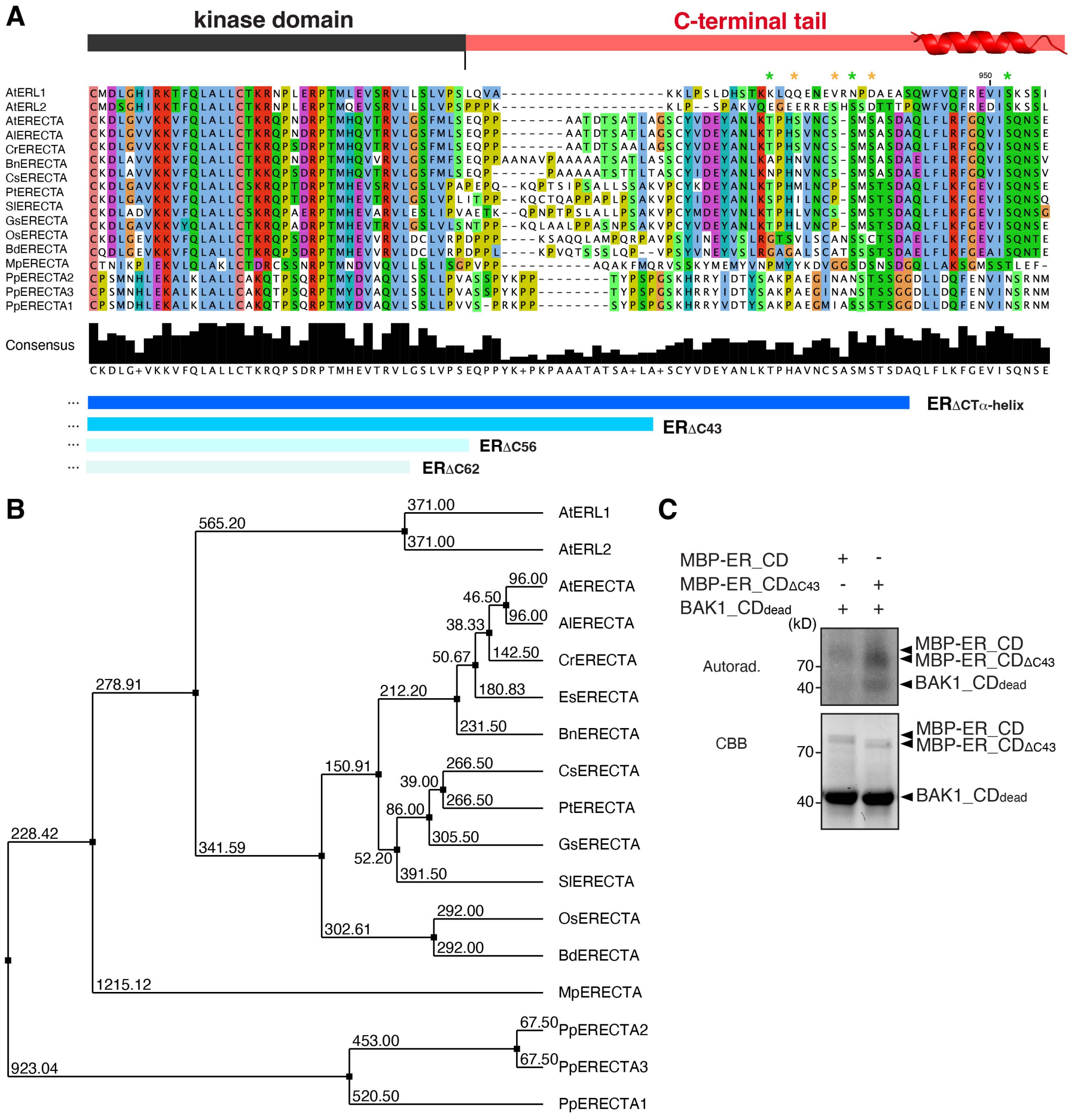

Fig. S1. The phylogenetic analysis of ER orthologs across land plants revealing sequence conservation of the C-terminal tail domain, and *in vitro* transphosphorylation of BAK1 by ER

(A) Amino-acid sequence alignments of the C-terminal tail domain of ER and its orthologs. Two *Arabidopsis* ER paralogs, ERL1 and ERL2, are also included for a comparison. The full-lengths amino-acid sequences of the following proteins were retrieved and subjected to alignment using Clustal W: *Arabidopsis thaliana* AtERL1 (AT5G62230.1), AtERL2 (AT5G07180.1), AtERECTA (AT2G26330.1), *Arabidopsis lyrata* AlERECTA (XP_020884443.1), *Capsella rubella* CrERECTA

(XP_006296065.1), *Brassica napus* BnERECTA (AGL91677.1), *Eutrema salsugineum* EsERECTA (XP_006408746.1), *Glycine soja* GsERECTA (KHN37058.1), *Solanus lycospersicum* SlERECTA (XP_004245043.1), *Poplus trichocarpa* PtERECTA (XP_024458576.1), *Oryza sativa cv japonica* OsERECTA (XP_015642019.1), *Brachypodium distachyon* BdERECTA (KQK19116.1), Marchantia polymorpha MpERECTA (Mapoly0070s0019.1), and *Physcomitrella patens* PpERECTA1 (Pp3c2_22410V3.1), PpERECTA2 (Pp3c1_17360V3.1), and PpERECTA3 (Pp3c21_9500V3.1). *In vivo* localized phosphorylation sites of ER found in this study are marked with green asterisks. Potential *in vivo* phosphorylation sites of ER are marked with orange asterisks. The three ER C-terminal deletion series are indicated with blue bars below.

(B) Molecular phylogenetic tree based on full-length amino-acid sequences of ER orthologs and paralogs using UPGMA (Unweighted Pair-Group Method using Arithmetic averages) calculated on the basis of PAM (percentage accepted mutations) 250, as a default parameter setting of Jalview (http://www.jalview.org/). A number at each node represents its associated branch length.

(C) Deletion of the C-terminal tail of ER_CD enhances its kinase activity. *In vitro* phosphorylation assays. BAK1_CD$_{dead}$ was used as a substrate, MBP-ER_CD or MBP-ER_CD$_{\Delta C43}$ were used as kinases in the presence of radiolabeled ATP. Autoradiography (Autorad.- upper) detects the extent of phosphorylation, and Coomassie Brilliant Blue (CBB) staining (lower) shows the protein loading.

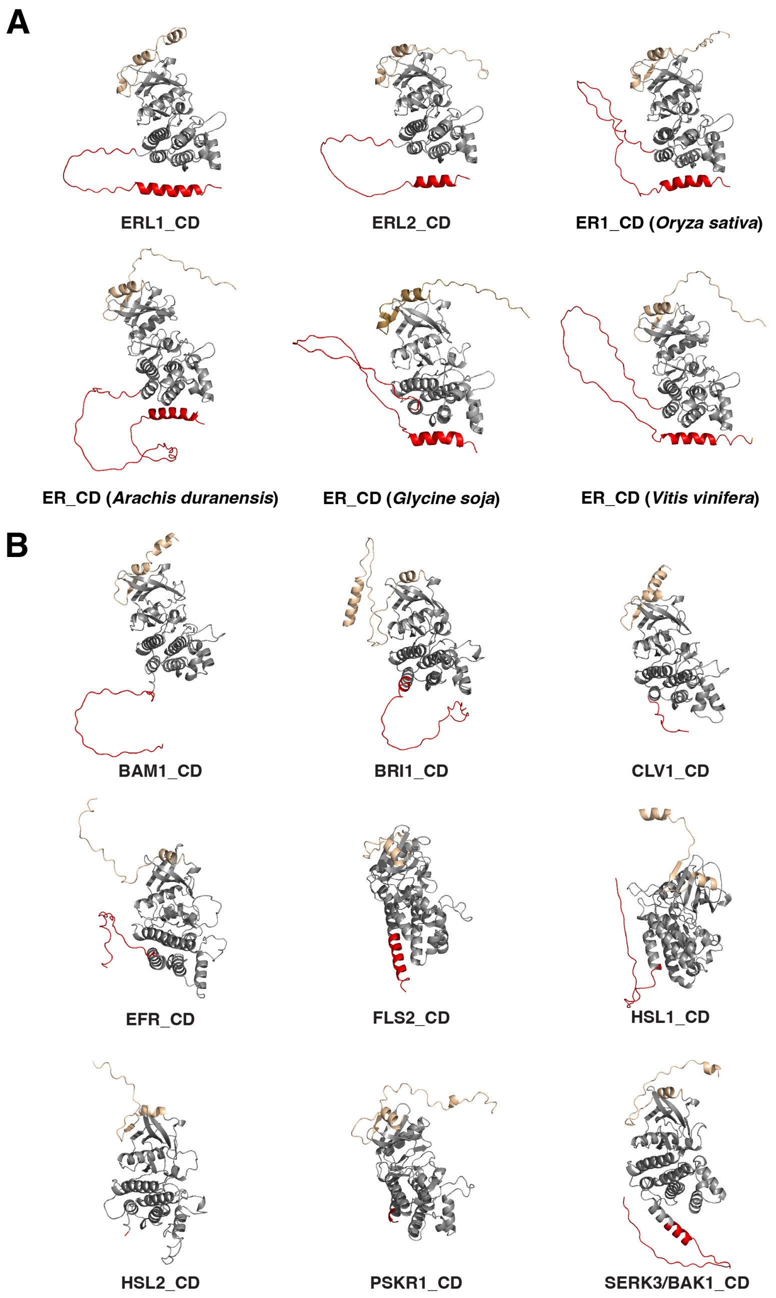

Fig. S2. Structural modeling of cytosolic domains of representative LRR-RKs

(A) Alphafold structural modeling of the cytosolic domain of Arabidopsis ERL1, ERL2, and ER orthologs from different species (*Oryza sativa*, *Arachis duranensis*, *Glycine soja*, and *Vitis vinifera*), with Juxtamembrane domain in sand, the Kinase domain in gray, and the C-terminal tail in red.

(B) Alphafold structural modeling of the cytosolic domain of Arabidopsis LRR-RKs BARELY ANY MERISTEM1 (BAM1), BRASSINOSTEROID INSENSTIVE1 (BRI1), CLAVATA1 (CLV1), EF-TU RECEPTOR (EFR), FLAGELLIN INSENSITIVE 2 (FLS2), HAESA-LIKE 1 (HSL1), HSL2, PHYTOSULFOKINE RECEPTOR1 (PSKR1), and SOMATIC EMBRYOGENESIS RECEPTOR KINASE3 (SERK3)/BRI1 ASSOCIATED KINASE1 (BAK1), with Juxtamembrane domain in sand, the Kinase domain in gray, and the C-terminal tail in red.

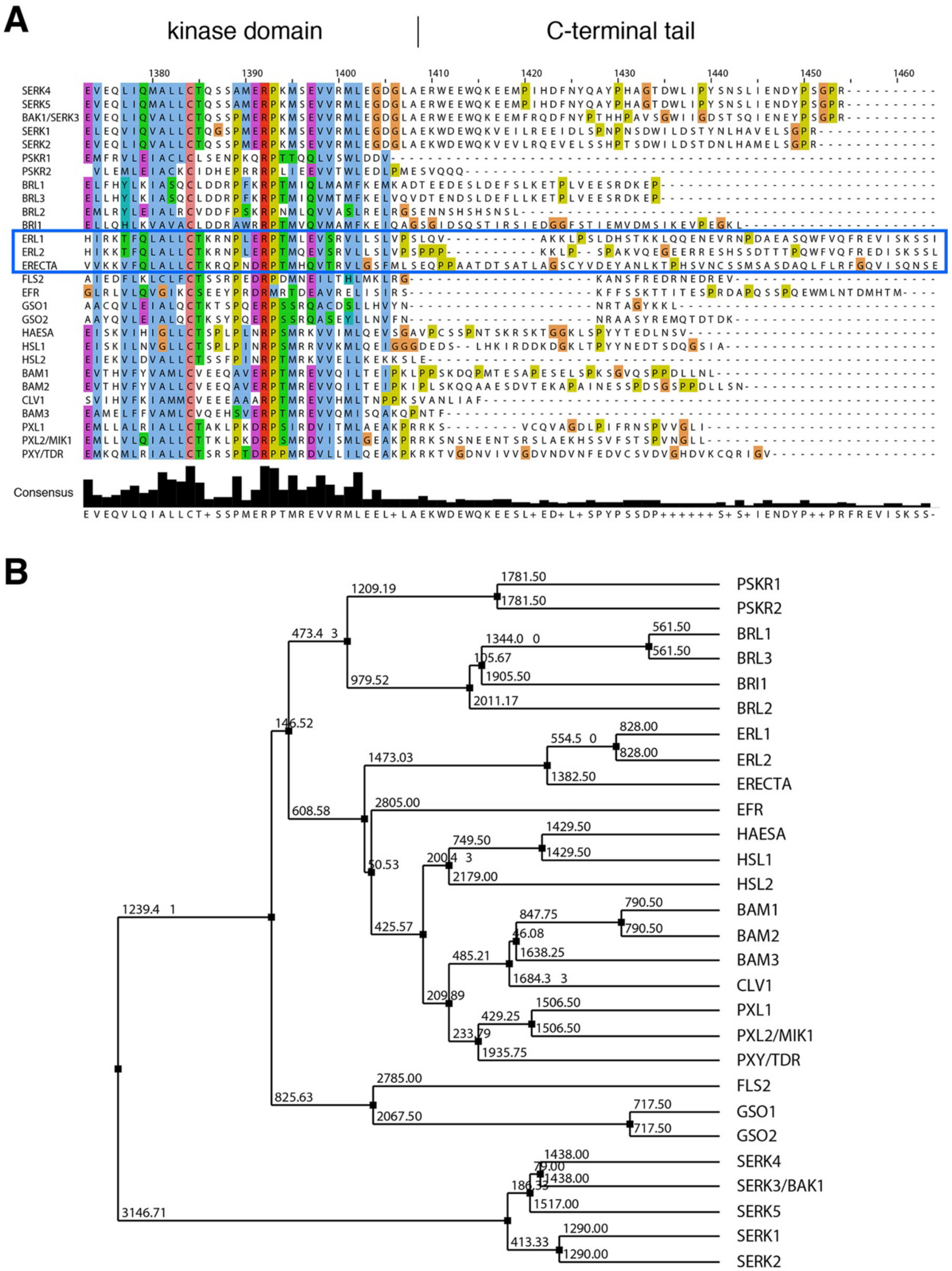

Fig. S3. The phylogenetic analysis of representative LRR-RKs revealing diversity in their C-terminal domains

(A) Amino-acid sequence alignments of the C-terminal tail domain of representative Arabidopsis LRR-RKs. The full-lengths amino-acid sequences of the following proteins were retrieved and subjected to alignment using Clustal W: BRI1, BRI1-LIKE1 (BRL1), BRL2, BRL3, BAM1, BAM2, CLV1, EFR, ER, ERL1, ERL2, FLS2, GASSHO1 (GSO1), GSO2, HAESA, HSL1, HSL2, PSKR1, PSKR2, PIXY/TDR (PXY), PIXY-LIKE1 (PXL1), PXL2, SERK1, SERK2, SERK3/BAK1, SERK4, and SERK5. Compared to the kinase domain, the C-terminal tail domains show little sequence conservation. Some subfamilies share conserved C-terminal domains (e.g., BRI1 family and SERK/BAK1 family). ER LRR-RK possesses a characteristic, very long C-terminal tail, which shares sequence similarity with its paralogs, ERL1 and ERL2 (highlighted with a blue bracket).
(B) Molecular phylogenetic tree based on full-length amino-acid sequences of representative Arabidopsis LRR-RKs using UPGMA (Unweighted Pair-Group Method using Arithmetic averages) calculated on the basis of PAM (percentage accepted mutations) 250, as a default parameter setting of Jalview (http://www.jalview.org/). A number at each node represents its associated branch length.

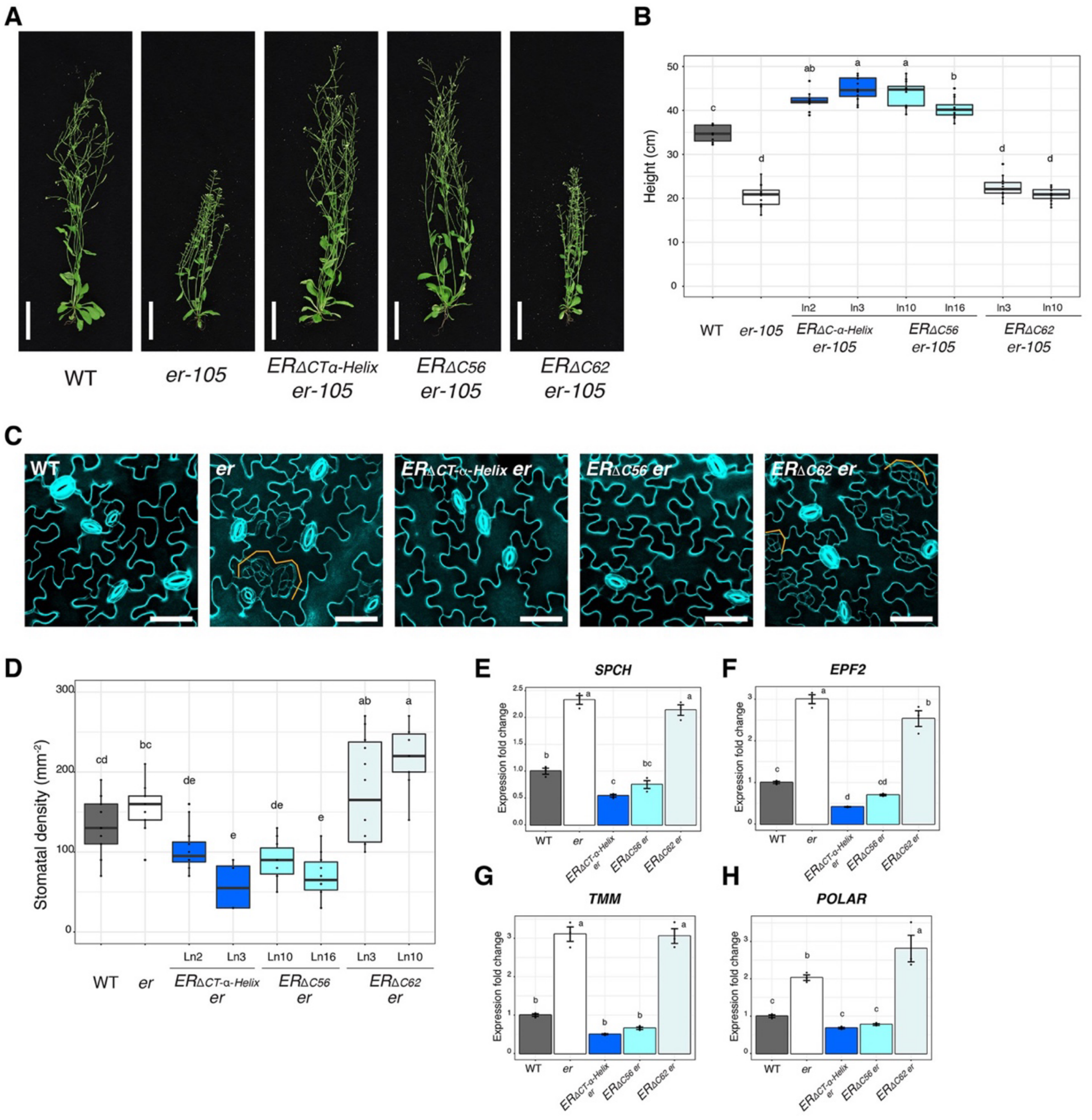

Fig. S4. Deletion of the C-terminal tail confers hyperactivity of ER in promoting inflorescence elongation and inhibiting stomatal development

(A) Representative plants of each genotype at 40 day-post-germination**.** Scale bars = 5 cm.

(B) Height of 40 day-post-germination (DPG40) wild-type (WT, n=11), *er* (n=11), $ER_{\Delta CT\alpha\text{-}Helix}$ *er* (n=12 for both line 2 and line 3), $ER_{\Delta C56}$ *er* (n=12 for both line 10 and line 16) and $ER_{\Delta C62}$ *er* (n=12 for line 3 and n=11 for line 10). One-way ANOVA followed by Tukey's HSD test was performed to categorize phenotypic groups.

(C) Representative confocal microscopy images of cotyledon abaxial epidermal at 10 day-post-germination (DPG10) of WT, *er*, $ER_{\Delta CT\alpha\text{-}Helix}$ *er***,** $ER_{\Delta C56}$ *er* and $ER_{\Delta C62}$ *er*. Excessive divisions of stomatal-lineage cells and clusters of SLGCs are indicated in orange. Scale bars = 50 µm.

(D) Stomatal density of DPG10 wild-type (WT, n=20), *er* (n=13), $ER_{\Delta CT\alpha\text{-}Helix}$ *er* (n=16 for line 2 and n=4 for line 3), $ER_{\Delta C56}$ *er* (n=10 for line 10 and line 16)**,** and $ER_{\Delta C62}$ *er* (n=16 for line 3 and n=10 for line 10). One-way ANOVA followed by Tukey's HSD test was performed to categorize phenotypic groups.

(E-H) RT-qPCR analysis of stomatal-lineage specific gene expression, *SPCH* (**E**), *EPF2* (**F**), *TMM* (**G**), and *POLAR* (**H**), from DPG3 seedlings of wild-type (WT), *er,* $ER_{\Delta CT\alpha\text{-}Helix}$ *er***,** $ER_{\Delta C56}$ *er* and $ER_{\Delta C62}$ *er.* Representative transgenic lines were used for each C-terminal deletion construct.

Transcript levels were normalized against *ACTIN* (*ACT2*) and adjusted to 1 for wild type. Bars, mean values of three technical replicates. Three biological replicates were performed. Error bars, s.e.m. One-way ANOVA followed by Tukey's HSD test was performed to categorize phenotypic groups.

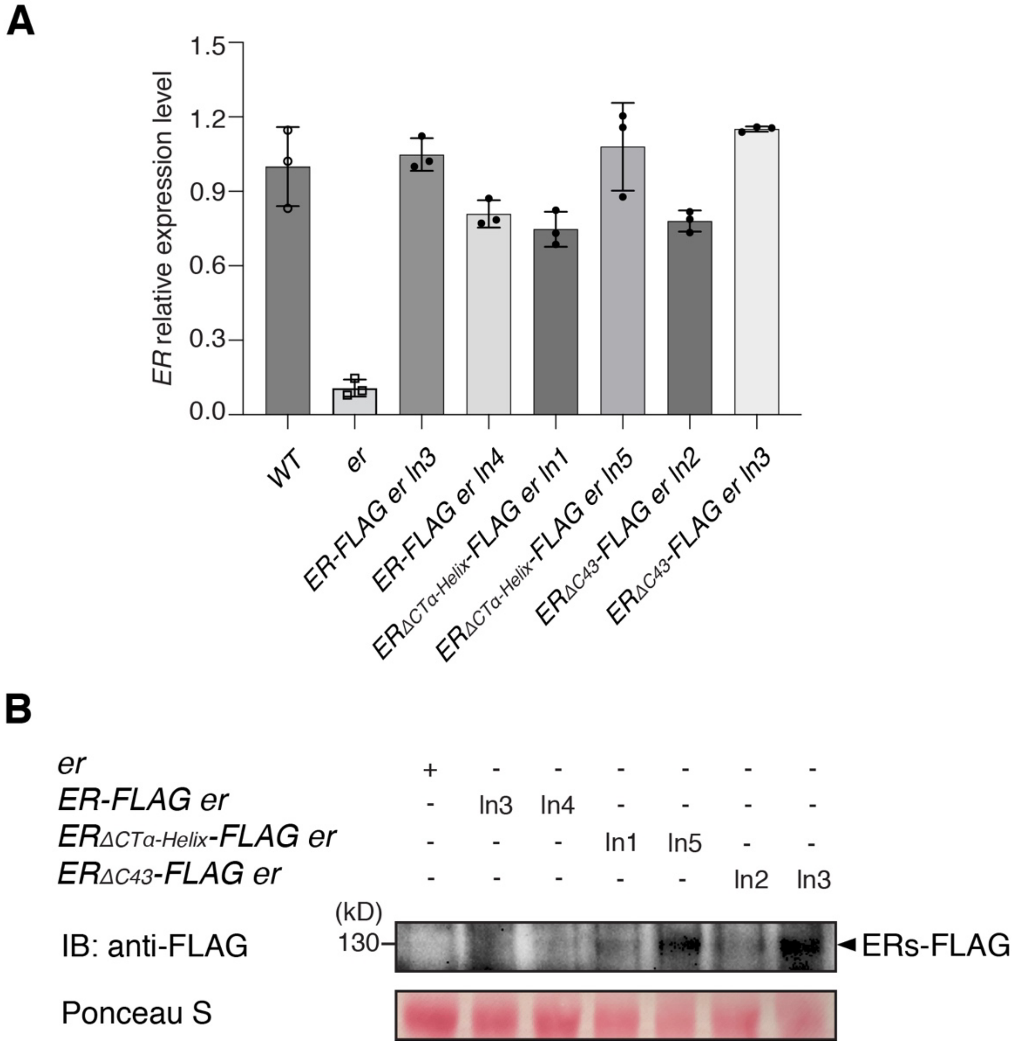

Fig. S5. Expression level of *ER* in *ER-FLAG er*, *ER*$_{\Delta CT\alpha\text{-}Helix}$ *er*, and *ER*$_{\Delta C43}$*-FLAG er*

(A) RT-qPCR analysis of *ER* in WT, *er, ER-FLAG er*, *ER*$_{\Delta CT\alpha\text{-}Helix}$*-FLAG er* and *ER*$_{\Delta C43}$*-FLAG er.* Transcript levels were normalized against *ACTIN* (*ACT2*) and adjusted to 1 for wild type. Bars, mean values of three technical replicates.

(B) Protein level of ER in WT, *er, ER-FLAG er*, *ER*$_{\Delta CT\alpha\text{-}Helix}$*-FLAG er*, and *ER*$_{\Delta C43}$*-FLAG er.* The immunoblots (IB) were probed with anti-FLAG antibody.

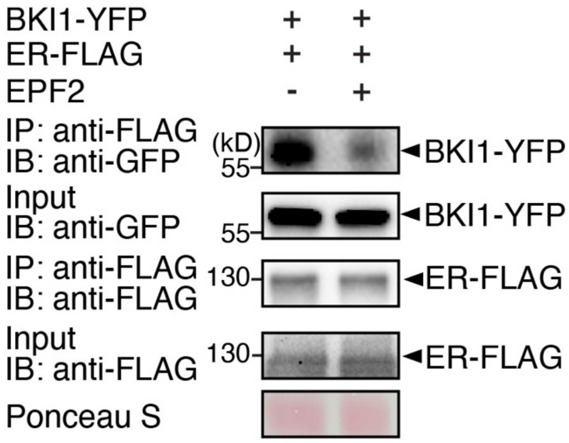

Fig. S6. EPF2 peptide perception decreases the association of ER with BKI1
After treatment with the MEPF2 peptide (EPF2), *ERpro::BKI1-YFP; ERpro::ER-FLAG* proteins expressed in transgenic plants were immunoprecipitated with anti-FLAG beads (IP), and the immunoblots (IB) were probed with anti-FLAG and anti-GFP antibodies, respectively.

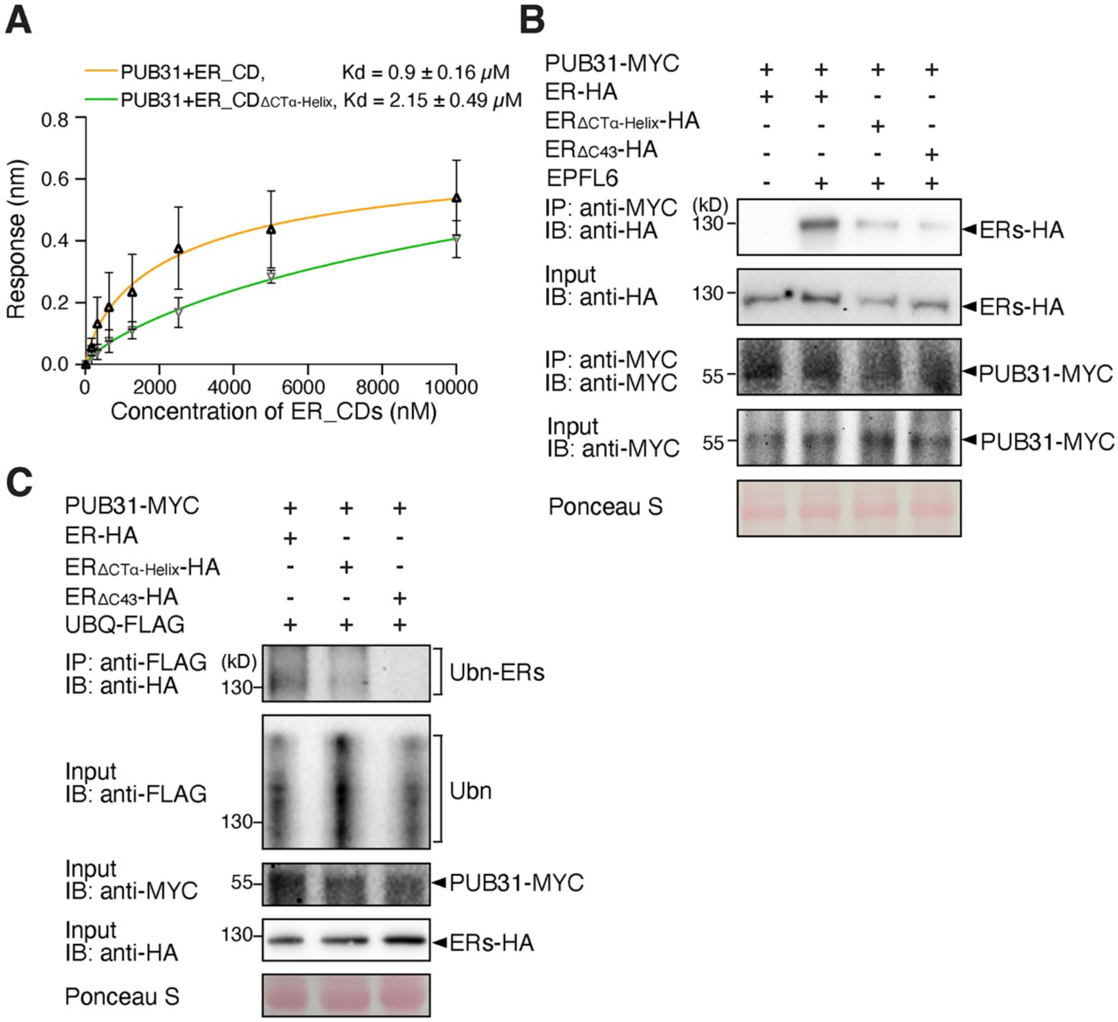

Fig. S7. The C-terminal tail is essential for the binding of ER with PUB31

(A) A quantitative analysis of interactions between PUB31 and ER_CD variants (ER_CD, $ER\_CD_{\Delta CT\alpha\text{-}Helix}$) using BLI. *In vitro* binding response curves for recombinantly purified GST-PUB31 and MBP-ER_CD variants at seven concentrations (156.25, 312.5, 625, 1,250, 2,500, 5,000, and 10,000 nM) are shown. Kd values are indicated. Data are representative of three independent experiments.

(B) Deletion of the C-terminal tail decreases the association of ER with PUB31 *in vivo*. Arabidopsis protoplasts were co-transfected with PUB31-MYC, together with ER-HA, $ER_{\Delta CT\alpha\text{-}Helix}$-HA, and $ER_{\Delta C43}$-HA. Five micromolar MEPFL6 peptide (EPFL6) was used for treatment for 1 hr. After immunoprecipitation using anti-MYC beads, the immunoblots (IB) were probed with anti-MYC and anti-HA antibodies, respectively.

(C) Deletion of the C-terminal tail decreases the ubiquitination of ER by PUB31 *in vivo.* Arabidopsis protoplasts were co-transfected with PUB31-MYC, FLAG-UBQ, together with ER-HA, $ER_{\Delta CT\alpha\text{-}Helix}$-HA, or $ER_{\Delta C43}$-HA. Five micromolar MEPFL6 peptide (EPFL6) was used for treatment for 1 hr. After immunoprecipitation using anti-FLAG beads, the ubiquitinated ER variants were probed with anti-HA antibody. The total ubiquitinated proteins were probed by anti-FLAG antibody and PUB31 proteins were probed by anti-MYC antibody. The inputs of ER were probed with anti-HA antibody.

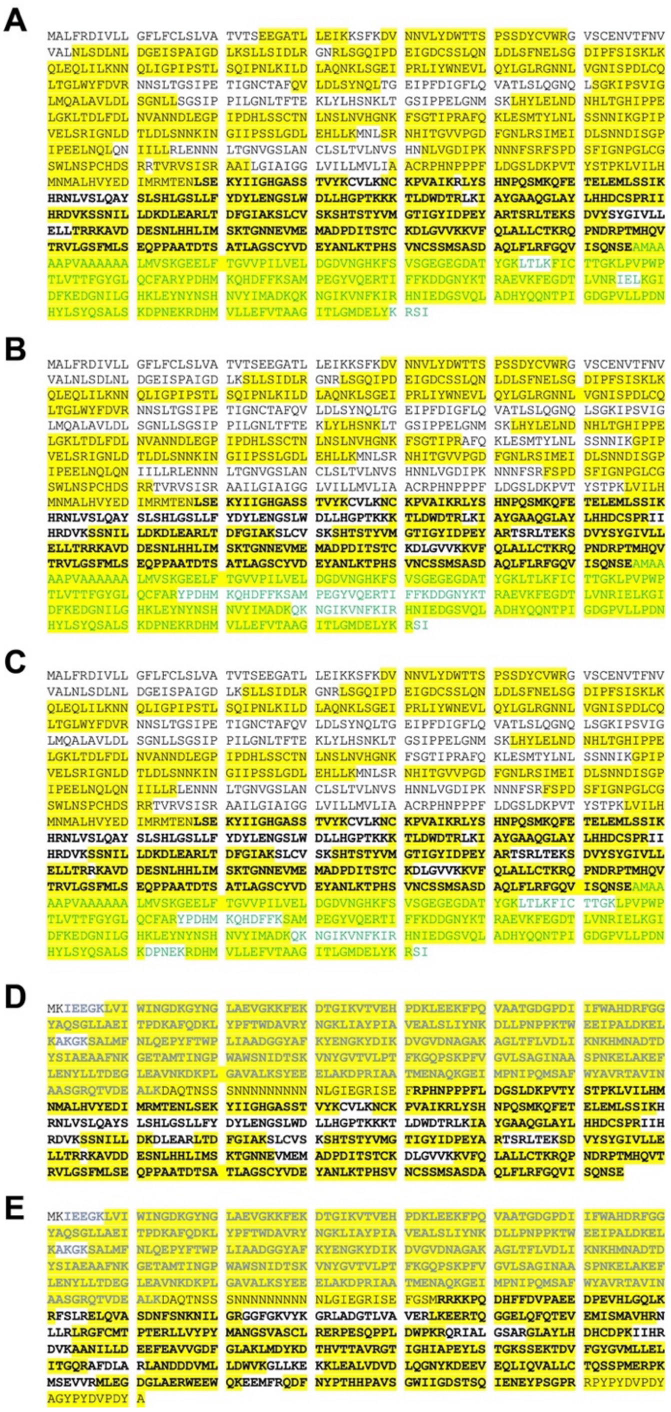

Fig. S8. Coverage of ER in the proteomics experiments

Total coverage of the ER-YFP protein in the different *in vivo* mass spectrometry experiments: Orbitrap Velos (**A**), Orbitrap Q Exactive (**B**), and Orbitrap Fusion (**C**). The identified sequence is highlighted in yellow. The cytoplasmic domain is marked in bold and YFP tag in green. (**D-E**). Coverage recombinant proteins in in vitro mass spectrometry experiments: MBP-ER_CD (**D**) and MBP-BAK1_CD (**E**). The identified sequence is highlighted in yellow. MBP-tag is marked in grey.

TPHSVNCSSM**Sp**ASDAQLFLR

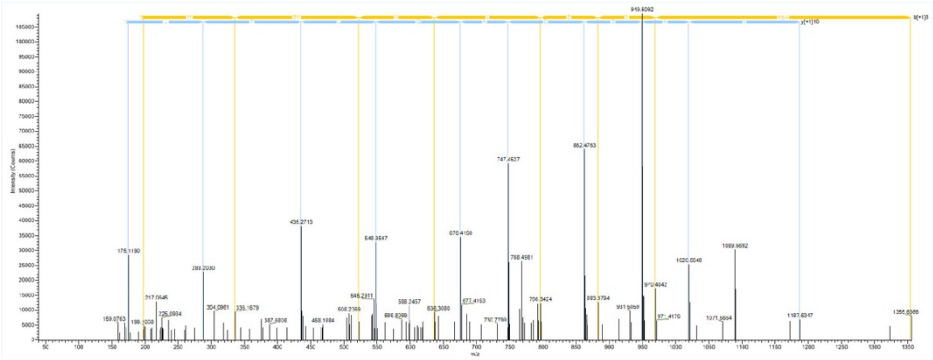

Fig. S9. Representative mass spectra for *in vitro* phosphorylation sites of ER in the tail
Representative site: Ser957. See Table S1 for experimental parameters.

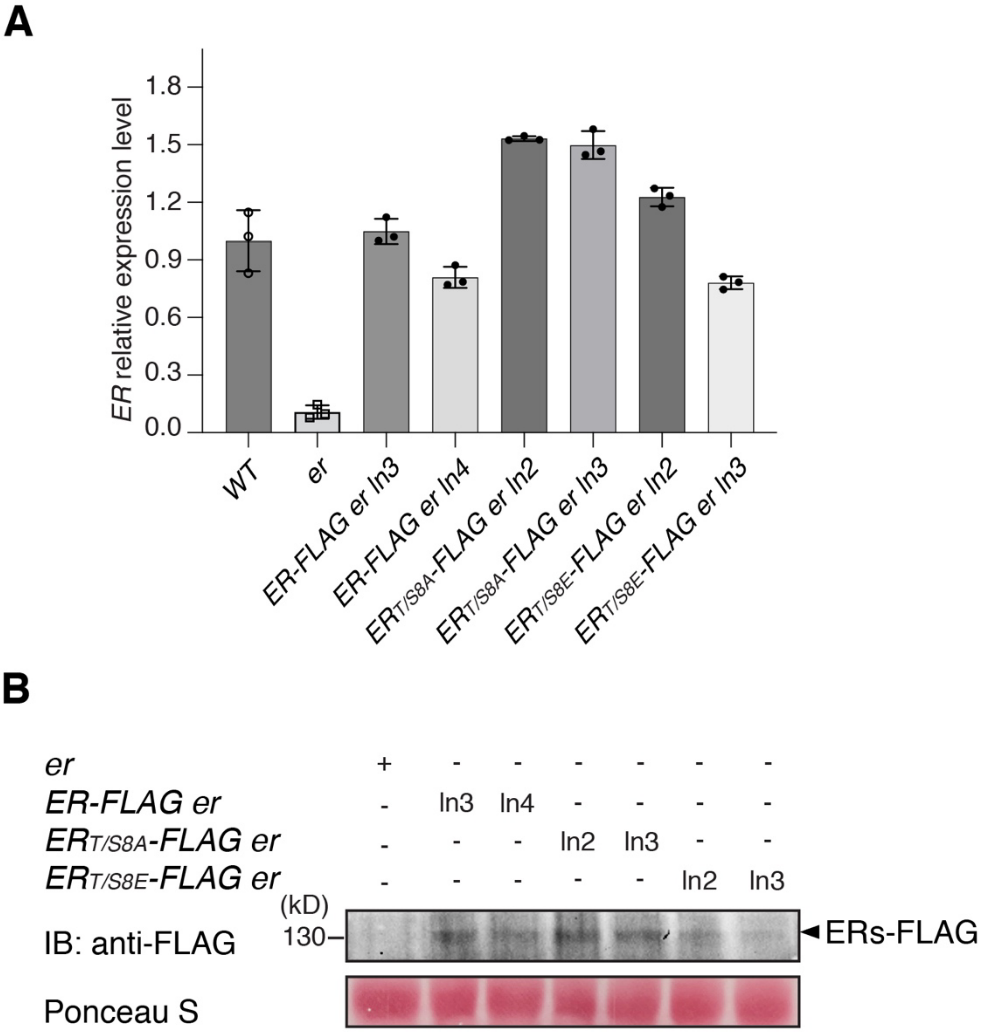

Fig. S10. Expression level of *ER* in *ER-FLAG er*, *ER*$_{T/S8A}$*-FLAG er*, and *ER*$_{T/S8E}$*-FLAG er*

(A) RT-qPCR analysis of *ER* in WT, *er, ER-FLAG er*, *ER*$_{T/S8A}$*-FLAG er*, and *ER*$_{T/S8E}$*-FLAG er* plants. Transcript levels were normalized against *ACTIN* (*ACT2*) and adjusted to 1 for wild type. Bars, mean values of three technical replicates.

(B) Protein level of ER in WT, *er, ER-FLAG er*, *ER*$_{T/S8A}$*-FLAG er*, and *ER*$_{T/S8E}$*-FLAG er* plants. The immunoblots (IB) were probed with anti-FLAG antibody.

A

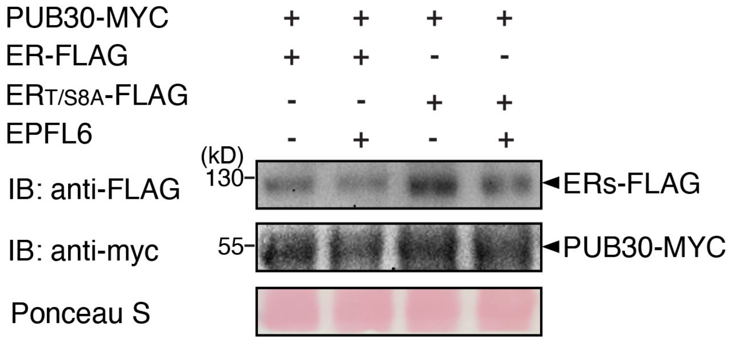

B

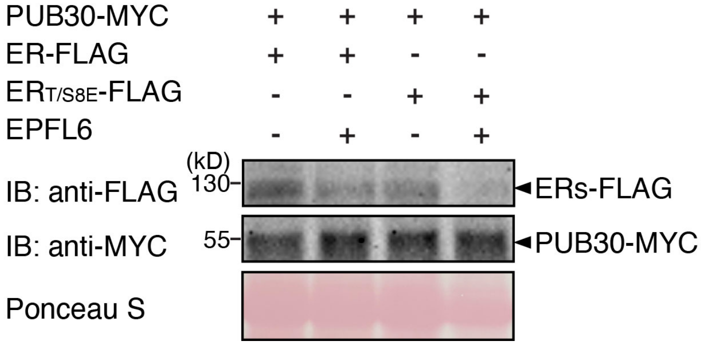

Fig. S11. Phosphorylation of ER_CT regulates ER protein stability
Shown are representative immunoblots of ER and its phosphorylation variants upon EPFL6 treatment. (**A**) Wild-type ER and phosphonull version; (**B**) Wild-type and phosphomimetic version in Arabidopsis protoplasts co-expressing PUB30-MYC. Protoplasts expressing the indicated proteins were treated with 50 μM CHX and 5 μM MEPFL6 for 3 hrs. before the total protein was examined with immunoblot. The experiment was repeated independently two times with similar results.

Table S1. Phosphorylation sites of ERECTA

bold p: phosphorylations on preceding Ser, Thr, Tyr
M*: oxidation of Met
C: all cysteine residues are carbamidomethylated

Table S1A: Phosphorylation sites of ERECTA *in vivo*

Protein region	Peptide sequence	Identified site	Theoretical *m/z*	Charge	Observed *m/z*	Mascot Score	Ascore/s	Site localization
678-687	LY**Sp**HNPQSMK	Ser680	642.7758	2	642.7760	25.91	33.98	localized
803-822	SHTST**Yp**VMGTIGYIDPEYAR	Tyr808	781.0117	3	781.0158	24.93	9.45	ambiguous
947-966	TPH**Sp**VNCSSM*SASDAQLFLR	Ser950	768.6643	3	768.6647	44.17	0	ambiguous
947-966	TPHSVNC**Sp**SMSASDAQLFLR	Ser954	763.3327	3	763.3343	57.02	0	ambiguous
947-966	TPHSVNCS**Sp**MSASDAQLFLR	Ser955	763.3327	3	763.3336	55.14	11.12	ambiguous
947-966	TPHSVNCSSM* **Sp**ASDAQLFLR	Ser957	768.6643	3	768.6694	52.81	0	ambiguous
967-995	FGQVI**Sp**QNSEAM*AAAAPVAAAAAALM*VSK	Ser972	963.1292	3	963.1296	55.79	0	ambiguous

Table S1B: Phosphorylation sites of ERECTA *in vivo*, repeat

Protein region	Peptide sequence	Identified site/s	Theoretical *m/z*	Charge	Observed *m/z*	Xcorr; deltaCn	Ascore/s	Site localization
127-139	EL**Sp**GDIPFSI**Sp**KL	Ser129 and Ser137	783.34863	2	783.3505	2.55; 0.125	60.76, 52.75	localized
750-768	IAYGAAQGLAYLHHDC**Sp**PR	Ser766	727.3296	3	727.3243	1.24; 0.091	15.71	localized
797-802	SLCV**Sp**K	Ser801	387.1668	2	387.16653	1.121; 0.149	83.50	localized
943-952	ANLK**Tp**PHSVN	Thr947	580.7766	2	580.776	1.765; 0.106	82.07	localized
947-966	TPHSVNCS**Sp**M*SASDAQLFLR	Ser955	768.6643	3	768.66595	2.733; 0	20.41	localized
965-978	LRFGQVI**Sp**QNSEAM*	Ser972	838.3791	2	838.3758	1.744; 0.195	41.33	localized

Table S1C: Phosphorylation sites of ERECTA *in vitro*

									Observed in			
Protein region	Peptide sequence	Identified site	Theoretical *m/z*	Charge	Observed *m/z*	Mascot Score	Ascore/s	Site localization	ER	ER kinase dead	ER + BAK1	ER kinase dead + BAK1
linker + 603-625	ISEFRPHNPPPFLDG**Sp**LDKPVTYSTPK	Ser614	780.6364	4	780.6396	14.03	40.4	localized			V	
677-687	L**Yp**SHNPQSMK	Tyr679	642.7758	2	642.7772	26.37	0	ambiguous			V	V
776-783	**Sp**SNILLDK	Ser776	485.2363	2	485.2364	45.39	17.01	localized			V	V
803-822	SH**Tp**STYVMGTIGYIDPEYAR	Thr805	1171.014	2	1171.0147	83.27	14.02	localized	V		V	V
803-822	SHT**Sp**TYVMGTIGYIDPEYAR	Ser806	1171.014	2	1171.0127	78.05	17.01	localized	V		V	V
847-862	AVDDE**Sp**NLHHLIMSK	Ser853	596.9377	3	596.9391	20.94	47.94	localized			V	
863-881	TGNNEVMEMADPDI**Tp**STCK	Thr877	1096.9309	2	1096.9347	56.79	14.02	localized			V	V
899-912	RQPNDRP**Tp**MHQVTR	Thr906	454.7163	4	454.7166	37.31	37.19	localized			V	V
913-946	VLGSFML**Sp**EQPPAATDTSATLAGSCYVDEYANLK	Ser920	1229.5596	3	1229.5602	61.35	0	ambiguous			V	V
947-966	TPH**Sp**VNCSSMSASDAQLFLR	Ser950	763.3327	3	763.3329	76.28	14.62	localized			V	V
947-966	TPHSVNCSSM* **Sp**ASDAQLFLR	Ser957	768.6643	3	768.6661	59.98	25.18	localized			V	V
967-976	FGQVI**Sp**QNSE	Ser972	594.7503	2	594.7519	12.48	0	ambiguous			V	

Table S2. Phosphorylation sites of BAK1

bold: phosphorylations on Ser or Thr
M*: oxidation of Met
c: carbamidomethylation of Cys

Protein region	Sequence	Identified site/s	Theoretical *m/z*	Charge	Observed *m/z*	Mascot Score	Ascore/s	site localization
273-288	RF**Sp**LRELQVASDNFSNK	Ser275	697.6721	3	697.6747	19.46	43.19	localized
278-288	ELQVA**Sp**DNFSNK	Ser283	716.3112	2	716.3139	27.8	49.79	localized
278-288	ELQVASDNF**Sp**NK	Ser287	477.8766	3	477.8783	21.85	31.3	localized
318-341	EER**Tp**QGGELQFQTEVEM*ISM*AVHR	Ser321	973.1001	3	973.1001	61.44	86.71	localized
318-341	EER**Tp**QGGELQFQ**Tp**EVEMISMAVHR	Ser321, Thr330	989.0922	3	989.0984	43.41	67.93, 69.38	localized
318-341	EER**Tp**QGGELQFQTEVEMI**Sp**MAVHR	Ser321, Ser336	989.0922	3	989.0941	30.78	34.01, 33.54	localized
322-314	TQGGELQFQ**Tp**EVEMI**Sp**MAVHR	Thr330, Ser336	1276.0415	2	1276.0451	25.16	59.64, 57.44	localized
348-356	GFcM*TPTpER	Thr354	597.7196	2	597.7209	23.31	30.83	localized
357-373	LLVYPYMANG**Sp**VAScLR	Ser367	997.4674	2	997.4710	39.84	41.96	localized
357-373	LLVYPYM*ANG**Sp**VA**Sp**cLR	Ser367, Ser370	1045.448	2	1045.4477	23.73	24.47, 42.72	localized
357-373	LLVYPYMANGSVA**Sp**cLR	Ser370	665.314	3	665.3160	24.57	23.62	localized
374-387	ERPE**Sp**QPPLDWPKR	Ser378	605.6244	3	605.6261	43.07	1,000.00	localized
390-396	IALG**Sp**AR	Ser394	384.1942	2	384.1947	27.44	1,000.00	localized
437-450	LMD**Yp**KD**Tp**HVTTAVR	Tyr440, Thr443	603.9263	3	603.9274	11.51	33.25, 10.92	localized
442-450	D**Tp**HVTTAVR	Thr443	540.2477	2	540.2493	27.23	37.81	localized
437-450	LMDYKD**Tp**HV**Tp**TAVR	Thr443, Thr446	905.3858	2	905.3881	17.43	20.75, 17.01	localized
437-465	LMDYKD**Tp**HV**TpTp**AVRG**Tp**IGHIAPEYLSTGK	Thr443, Thr446, Thr447, Thr452	1165.5017	3	1165.5029	22.15	20.05, 22.49, 23.43, 29.81	localized
442-450	DTHV**TpTp**AVR	Thr446, Thr447	580.2309	2	580.2314	14.85	37.54, 31.27	localized
451-465	G**Tp**IGHIAPEYLSTGK	Thr452	812.3925	2	812.3922	52.9	68.8	localized
451-465	GTIGHIAPE**Yp**LSTGK	Tyr460	812.3925	2	812.3937	67.77	48.87	localized
451-465	GTIGHIAPEYL**SpTp**GK	Ser462, Thr463	568.5862	3	568.5878	18.36	25.97, 25.97	localized
553-558	M***Sp**EVVR	Ser554	408.6697	2	408.6702	26.37	1,000.00	localized
580-612	QDFNYPTHHPAVSGWIIGD**Sp**TSQIENEYPSGPR	Ser599	945.6745	4	945.6772	62.4	22.34	localized

Table S3. List of plasmids used in this study

Plasmid ID	Insert	Backbone vector	Entry clones	Purpose	Notes
pKUT196	ERpro::gER	pPZP222	Spec/Strep	For plant transformation	This study
pKUT603	ERpro::gERΔCTα-Helix	pPZP222	Spec/Strep	For plant transformation	This study
pKUT604	ERpro::gERΔC56	pPZP222	Spec/Strep	For plant transformation	This study
pKUT605	ERpro::gERΔC62	pPZP222	Spec/Strep	For plant transformation	This study
pCLL202	ERpro::gER-FLAG	R4pGWB410	Spec	For plant transformation	This study
pCLL261	ERpro::gERΔCTα-Helix-FLAG	R4pGWB410	Spec	For plant transformation	This study
pCLL262	ERpro::gERΔC43-FLAG	R4pGWB410	Spec	For plant transformation	This study
pJM284	ERpro::ER-YFP	pPZP222	Spec/Strep	For plant transformation	This study
pLGR3	ERpro::ERK676E-YFP	R4pGWB540	Spec	For plant transformation	This study
pCLL265	ERpro::BKI1-YFP	R4pGWB540	Spec	For plant transformation	This study
pCLL124	PUB30pro::PUB30-YFP	R4pGWB540	Spec	For plant transformation	Chen et al., (2023)
pCLL126	PUB31pro::PUB31-YFP	R4pGWB540	Spec	For plant transformation	Chen et al., (2023)
pCLL272	ERpro::gERT/S8A-FLAG; gERT/S8A, aa NO. 947, 950, 954, 955, 957, 959, 972,975 from Ser/Thr. to Ala.	R4pGWB410	Spec	For plant transformation	This study
pCLL273	ERpro::gERT/S8E-FLAG; gERT/S8E, aa NO. 947, 950, 954, 955, 957, 959, 972,975 from Ser/Thr. to Glu.	R4pGWB410	Spec	For plant transformation	This study
pJSL104	gER	pENTR/D-topo	Kan	As entry clone	This study
pLGR1	ER promotor	pENTR/5'-topo	Kan	As entry clone	Lee et al. (2012)
pCLL257	gERΔCTα-Helix	pENTR/D-topo	Kan	As entry clone	Lee et al. (2012)
pCLL258	gERΔC43	pENTR/D-topo	Kan	As entry clone	This study
pCLL259	gERΔC56	pENTR/D-topo	Kan	As entry clone	This study
pCLL260	gERΔC62	pENTR/D-topo	Kan	As entry clone	This study
pCLL264	BKI1	pENTR/D-topo	Kan	As entry clone	This study
pCLL266	gERS2A, aa NO. 972,975 from Ser. to Ala.	pENTR/D-topo	Kan	As entry clone	This study
pCLL267	gERS2E, aa NO. 972,975 from Ser. to Glu.	pENTR/D-topo	Kan	As entry clone	This study
pCLL268	gERT/S8A, aa NO. 947, 950, 954, 955, 957, 959, 972,975 from Ser/Thr. to Ala.	pENTR/D-topo	Kan	As entry clone	This study
pCLL269	gERT/S8E, aa NO. 947, 950, 954, 955, 957, 959, 972,975 from Ser/Thr. to Glu.	pENTR/D-topo	Kan	As entry clone	This study
pJA51	MBP-ER_CD	pMAL-c2	Amp	For recombination expression	Chen et al., (2023)
pJA69	MBP-ER_CDΔC43	pMAL-c2	Amp	For recombination expression	This study
pKUT537	MBP-ER_CDK676E	pMAL-c2	Amp	For recombination expression	This study
pMM159	MBP-ER_CDΔCTα-Helix	pMAL-c2	Amp	For recombination expression	This study
pCLL276	MBP-ER_CDT/S8A, aa NO. 947, 950, 954, 955, 957, 959, 972,975 from Ser/Thr. to Ala.	pMAL-c2	Amp	For recombination expression	This study
pCLL277	MBP-ER_CDT/S8E, aa NO. 947, 950, 954, 955, 957, 959, 972,975 from Ser/Thr. to Glu.	pMAL-c2	Amp	For recombination expression	This study
pCLL278	BKI1-CDS	pGEX4T-1	Amp	For recombination expression	This study
pCLL107	PUB30-CDS	pGEX4T-1	Amp	For recombination expression	Chen et al., (2023)
pCLL109	PUB31-CDS	pGEX4T-1	Amp	For recombination expression	Chen et al., (2023)
pJSL68	mature EPF2	pBADgΠI	n/a	For recombination expression	Lee et al. (2012)
pJSL79	mature EPFL6	pBADgΠI	n/a	For recombination expression	Uchida et al. (2012)
pJSL84	ERECTA without stop codon (1-5526bp)-2xHA	pHBT	n/a	For protoplast transfection	Lee et al. (2012)
pCLL148	PUB30 without stop codon (1- 1344bp)-1xMYC	pHBT	n/a	For protoplast transfection	Chen et al., (2023)
pCLL149	PUB31 without stop codon (1-1332bp)-1xMYC	pHBT	n/a	For protoplast transfection	Chen et al., (2023)
pCLL279	gERΔCTα-Helix-2xHA	pHBT	n/a	For protoplast transfection	This study
pCLL282	gERΔC43-2xHA	pHBT	n/a	For protoplast transfection	This study
pJSL85	gER-2xFLAG	pHBT	n/a	For protoplast transfection	This study
pPFB51	gERT/S8A (aa NO. 947, 950, 954, 955, 957, 959, 972,975 from Ser/Thr. to Ala.)-2xFLAG	pHBT	n/a	For protoplast transfection	This study
pPFB52	gERT/S8E (aa NO. 947, 950, 954, 955, 957, 959, 972,975 from Ser/Thr. to Glu.)-2xFLAG	pHBT	n/a	For protoplast transfection	This study
pCLL280	gERΔCTα-Helix-2xFLAG	pHBT	n/a	For protoplast transfection	This study
pCLL281	gERΔC43-2xFLAG	pHBT	n/a	For protoplast transfection	This study
	FLAG-UBQ	pHBT	n/a	For protoplast transfection	Zhou et al. (2018)

Table S4. List of oligo DNA used in this study

Gene names	Primer names	Sequences (5' to 3')	Note
**For Constructs**			
ERECTA	ERg4359.f	CAACAATGATCTGGAAGGAC	to make pKUT603/4/5
	ERg7123stopB.rc	CGGGATCCTTAGAGAACACGAGTCACCTGGT	to make pKUT605
	ERg7141stopB.rc	CGGGATCCTTACGATAGCATAAAACTGCCGA	to make pKUT604
	ERg7263stopB.rc	CGGGATCCTTAAGCATCAGAAGCACTCATGGA	to make pKUT603
	JA28-ER T947E.f	GTATGCAAATCTCAAGGAGCCTCATTCTGTC	For mutagenesis of T947E in ERECTA
	JA29-ER T947E.rc	GACAGAATGAGGCTCCTTGAGATTTGCATAC	For mutagenesis of T947E in ERECTA
	JA30-ER T947A.f	GTATGCAAATCTCAAGGCTCCTCATTCTGTC	For mutagenesis of T947A in ERECTA
	JA31-ER T947A.rc	GACAGAATGAGGAGCCTTGAGATTTGCATAC	For mutagenesis of T947A in ERECTA
	JA36-ER S972E.f	GGTTTGGACAAGTTATTGAGCAGAACAGTGAGTAG	For mutagenesis of S972E in ERECTA
	JA37-ER S972E.rc	CTACTCACTGTTCTGCTCAATAACTTGTCCAAACC	For mutagenesis of S972E in ERECTA
	JA38-ER S972A.f	GGTTTGGACAAGTTATTGCTCAGAACAGTGAGTAG	For mutagenesis of S972A in ERECTA
	JA39-ER S972A.rc	CTACTCACTGTTCTGAGCAATAACTTGTCCAAACC	For mutagenesis of S972A in ERECTA
	MM52-ER S954A/S955A.fwd	CTGTCAATTGCGCCGCCATGAGTGCTTCTG	For mutagenesis of S954A/S955A in ERECTA
	MM53-ER S954A/S955A.rc	CAGAAGCACTCATGGCGGCGCAATTGACAG	For mutagenesis of S954A/S955A in ERECTA
	MM36-ER S954E.fwd	CTGTCAATTGCGAGTCCATGAGTGCTTCTG	For mutagenesis of S954E in ERECTA
	MM37-ER S954E.rc	CAGAAGCACTCATGGACTCGCAATTGACAG	For mutagenesis of S954E in ERECTA
	MM89-ER S954E/S955E.fwd	CTGTCAATTGCGAGGAGATGAGTGCTTCTG	For mutagenesis of S954E/S955E in ERECTA
	MM90-ER S954E/S955E.rc	CAGAAGCACTCATCTCCTCGCAATTGACAG	For mutagenesis of S954E/S955E in ERECTA
	gER-Dtopo-LP	caccATGGCTCTGTTTAGAGATATTGT	for gERs-D-TOPO
	gER-Dtopo-RP	CTCACTGTTCTGAGAAATAACTTG	for gER-D-TOPO
	gERΔCTα-Helix-Dtopo-RP	AGCATCAGAAGCACTCATGGAAG	for gERΔCTα-Helix-D-TOPO
	gERΔC43-Dtopo-RP	CAGCGTCGCTGACGTGTCAGTC	for gERΔC43-D-TOPO
	gERΔC56-Dtopo-RP	CGATAGCATAAAACTGCCGAG	for gERΔC56-D-TOPO
	gERΔC62-Dtopo-RP	GAGAACACGAGTCACCTGGTGC	for gERΔC62-D-TOPO
	gER-Cnull-Dtopo-RP	CTCAGCGTTCTGAGCAATAACTTGT	for gERS2A-TOPO and gERT/S8A-TOPO
	gER-Cmimic-Dtopo-RP	CTCTTCGTTCTGCTCAATAACTTGT	for gERS2E-TOPO and gERT/S8E-TOPO
	BKI1-Dtopo-LP	caccATGGAAACTAATCTACAACAG	for BKI1-D-TOPO
	BKI1-Dtopo-RP	AGAATCCTTAACCTTATCAT	for BKI1-D-TOPO
	MBP-ER_CD-BamH1-LP	GAAGGATTTCAGAATTCGGATCCGCTTGCCGACCGCATAATCCTC	for MBP-ER_CDΔCTα-Helix
	MBP-ER_CDΔCTα-Helix-Sal1-RP	GCCAAGCTTGCCTGCAGGTCGACCTAAAACAGTTGAGCATCAGAAGCAC	for MBP-ER_CDΔCTα-Helix
	MBP-ER_CD-EcoR1-LP	CACGAATTCCGACCGCATAATCCTCCTCC	for MBP-ER_CDΔC43
	MBP-ER_CDΔC43-Sal1-RP	CACGTCGACTTAGCGTCGCTGACGTGTCAG	for MBP-ER_CDΔC43
	MBP-ER (919-976)-BamH1-LP	GAAGGATTTCAGAATTCGGATCCCTATCGGAACAACCACCTGCT	for MBP-ER_C-tail
	MBP-ER (919-976)-Sal1-RP	GCCAAGCTTGCCTGCAGGTCGACCTACTCACTGTTCTGAGAAAT	for MBP-ER_C-tail
	ERCD-mid-F (for T/S8A, T/S8E)	ATAATGTCAAAGACGGGGAAC	for MBP-ER_CDT/S8A, T/S8E
	ERCD-mid-R (for T/S8A, T/S8E)	GTTCCCCGTCTTTGACATTAT	for MBP-ER_CDT/S8A, T/S8E
	pHBTFLAG/HA-ER-infusBamH1-F	GCTCTCGGCTCCCTCTCCCCTTGCTCCGTGGATCCATGGCTCTGTTTA	for pHBT-ERs-HA/FLAG
	pHBTHA-ERΔCTα-Helix-infuStu1-R	CCAGCGTAGTCTGGAACGTCGTATGGGTAAGGCCTAGCATCAGAAGCA	for pHBTHA-ERΔCTα-Helix
	pHBTFLAG-infuStu1_ΔCTα-Helix-R	GTCACTTGTCATCGTCGTCCTTGTAGTCAGAAGGCCTAGCATCAGAAGC	for pHBTFLAG-gERΔCTα-Helix
	pHBTFLAG-infuStu1_ΔC43-R	GTCACTTGTCATCGTCGTCCTTGTAGTCAGAAGGCCTCAGCGTCGCTG	for pHBTFLAG-gERΔC43
**For genotyping**			
	ERg2248	AAGAAGTCATTCAAAGATGTGA	ERg2248+ERg3016rc for WT band
	ERg3016rc	AGAATTTCCAGGTTTGGAATCTGT	
	er-105rc	AGCTGACTATACCCGATACTGA	ERg2248+er-105rc for insert band
**For q-RT PCR**			
	ACT2-F	TCATCTTCTTCCGCTCTTTCTT	
	ACT2-R	AATCCAGCCTTCACCATACC	
	qERECTA-F	ACAGCTTAAATGTTCATGG	
	qERECTA-R	GGACCTTTGATATTGTTGC	

SI References

1. L. Chen *et al.*, Direct attenuation of Arabidopsis ERECTA signalling by a pair of U-box E3 ligases. *Nat Plants* **9**, 112-127 (2023).
2. T. Nakagawa *et al.*, Development of R4 gateway binary vectors (R4pGWB) enabling high-throughput promoter swapping for plant research. *Biosci Biotechnol Biochem* **72**, 624-629 (2008).
3. J. S. Lee *et al.*, Direct interaction of ligand–receptor pairs specifying stomatal patterning. *Genes Dev* **26**, 126-136 (2012).
4. J. S. Lee *et al.*, Competitive binding of antagonistic peptides fine-tunes stomatal patterning. *Nature* **522**, 439-443 (2015).
5. N. Uchida *et al.*, Regulation of inflorescence architecture by intertissue layer ligand-receptor communication between endodermis and phloem. *Proc Natl Acad Sci U S A* **109**, 6337-6342 (2012).
6. D. Lu *et al.*, A receptor-like cytoplasmic kinase, BIK1, associates with a flagellin receptor complex to initiate plant innate immunity. *Proc Natl Acad Sci U S A* **107**, 496-501 (2010).
7. T. N. Uehara *et al.*, Casein kinase 1 family regulates PRR5 and TOC1 in the Arabidopsis circadian clock. *Proc Natl Acad Sci U S A* **116**, 11528-11536 (2019).
8. K. W. Bender *et al.*, Autophosphorylation-based Calcium (Ca2+) Sensitivity Priming and Ca2+/Calmodulin Inhibition of *Arabidopsis thaliana* Ca2+-dependent Protein Kinase 28 (CPK28). *J Biol Chem* **292**, 3988-4002 (2017).
9. S. K. Han *et al.*, MUTE Directly Orchestrates Cell-State Switch and the Single Symmetric Division to Create Stomata. *Dev Cell* **45**, 303-+ (2018).
10. A. Micsonai *et al.*, BeStSel: a web server for accurate protein secondary structure prediction and fold recognition from the circular dichroism spectra. *Nucleic Acid Res* **46**, W315-W322 (2018).
